# LiDAR-Based Road Surface Damage Classification: A Survey

**DOI:** 10.3390/s26082338

**Published:** 2026-04-10

**Authors:** Trevor Greene, Meisam Shayegh Moradi, Muhammad Umair, Nafiul Nawjis, Naima Kaabouch, Timothy Pasch

**Affiliations:** 1 Artificial Intelligence Research (AIR) Center, University of North Dakota, Grand Forks, ND 58202, USAumair.muhammad@ndus.edu (M.U.);; 2 Communications Department, University of North Dakota, Grand Forks, ND 58202, USA

**Keywords:** LiDAR, pavement distress, road surface damage, mobile laser scanning, point clouds, survey

## Abstract

Unlike image-only systems that falter in shadows, glare, and low contrast, LiDAR directly records surface geometry and supports depth-aware quantification. This survey examines LiDAR-based road surface damage classification across the entire pipeline, encompassing acquisition with mobile and terrestrial laser scanning, preprocessing and representation choices, supervised, semi-supervised, and unsupervised learning techniques, as well as multisensor fusion at early, mid, and late stages. A consistent thread is measurement, not just detection: we describe how LiDAR damage classification maps to agency practices such as the Distress Identification Manual and the Pavement Condition Index. We summarize datasets and evaluation protocols for detection, segmentation, 3D reconstruction, and ride quality. We outline practical concerns for corridor-scale deployment: calibration and timing, intensity normalization, tiling/streaming, and runtime budgeting. The review concludes with open problems and outlines directions for robust, severity-aware, and scalable field systems.

## 1. Introduction

Road surfaces deteriorate under the combined effects of traffic loading, moisture, freeze–thaw cycles, and material aging, leading to cracks, potholes, rutting, and other defects that increase safety risks and maintenance costs [1,2,3,4]. Visual inspection is fragile due to illumination, shadows, and texture differences that confound image-only systems, and unsealed roads introduce corrugation and localized settlement that fall outside simple “crack/pothole” cases [1,2,3,4]. Light detection and ranging (LiDAR) mitigates many of these pitfalls by capturing surface geometry with much less dependence on ambient lighting. Mobile and terrestrial laser scanning provide dense point clouds from which rut profiles, pothole depth and volume, and subtle surface defects can be measured precisely; depth anomalies that may be invisible in standard optical red, green, blue (RGB) images appear as height deviations in LiDAR [5,6,7,8,9,10,11,12,13]. When imagery is available, disciplined fusion enables LiDAR to provide scale and structure to the analysis. In comparison, images provide texture cues, improving detection in low-contrast or shadowed scenes and in remote/hard-to-access areas [2,3,4,5,6,7,8,9,10,11,12,13,14]. Despite strong convolutional neural network (CNN) results on RGB benchmarks, sensitivity to lighting, occlusion, season, and road type continues to motivate LiDAR-centric approaches and geometry-aware fusion [2,3,9,13,15,16,17,18,19,20,21,22].

The scope and contributions of this paper are as follows:

- **LiDAR-first pipeline:** We review the end-to-end 3D geometry workflow, from mobile laser scanning (MLS)/terrestrial laser scanning (TLS) acquisition and time-gridding through range images, 2.5D height/depth maps, and native point/voxel/graph learning, positioning LiDAR as the backbone, with other sensor fusion as an optional complement when alignment is reliable [2,3,9,13,14,21,22,23,24,25,26].
- **Label-efficiency strategies:** We consolidate three practical families for corridor-scale data that reduce annotation cost: (i) geometry-driven grouping without labels, (ii) reconstruction-based anomaly mapping, and (iii) semi-supervised learning that leverages small labeled subsets or weak labels, including region growing on MLS and competitive reconstruction networks [10,18,27,28,29].
- **Fusion design and reporting:** We compare early/mid/late fusion, treating extrinsic calibration and time synchronization as first-order determinants of realized gains. We highlight simple, reproducible reporting practices, including identical splits, single-stream baselines, and both per-stream and fused scores, to reveal the true fusion benefit rather than introducing ambiguity [2,9,13,21,22,26].
- **Actionable metrology:** We relate outputs to severity-linked measures, including crack width, length, and continuity, pothole depth and volume, and rut profiles, and map these quantities to agency frameworks such as the Distress Identification Manual (DIM) and the Pavement Condition Index (PCI), with attention to units, detrending, and reference-surface fitting to surrounding intact pavement for depth and volume estimation [2,4,6,7,30,31].
- **Coverage and boundaries:** Coverage runs through 2025, including graph neural networks for 3D crack topology, point-set models, transformer-based 2D/2.5D encoders, and recent low-cost and mobile LiDAR studies, alongside new datasets such as RSRD-Seg (2024) and a handheld iPhone-LiDAR cracks dataset (2025) [8,32,33,34,35,36,37]. Subsurface sensing (e.g., ground penetrating radar) and non-LiDAR-only modalities are out of scope unless LiDAR remains the primary signal.
- **Evaluation, resources, and deployment framing:** We summarize datasets, annotation practices, and evaluation metrics for detection, inventory, and severity estimation, and relate measurement outputs to DIM and PCI-oriented decision contexts. We also review the main deployment constraints identified in the literature, including labeling cost, domain and seasonal shift, calibration drift, robustness on unsealed roads, and runtime limits.

The literature consistently distinguishes between quick, binary screening and severity-aware inventory. LiDAR’s value is not only in finding distress but also in measuring it, so organizing tasks by intended use keeps analysis aligned with DIM/PCI and field decisions [5,8,13,30,31,38]. Preprocessing converts raw LiDAR point clouds into analysis-ready road data by aligning timestamps and coordinate frames and partitioning the corridor into overlapping tiles (typically 10–20%) to preserve elongated defects. Subsequent steps commonly include road surface extraction, noise removal, and normalization of sampling density and intensity. These operations aim to improve geometric consistency and reduce artifacts while preserving defect-relevant structure for downstream detection and measurement.

Table 1 summarizes the task–output–representation–evaluation crosswalk used in this review, linking each pavement-analysis task to its typical outputs, data representations, and evaluation focus.

**Figure 1:**
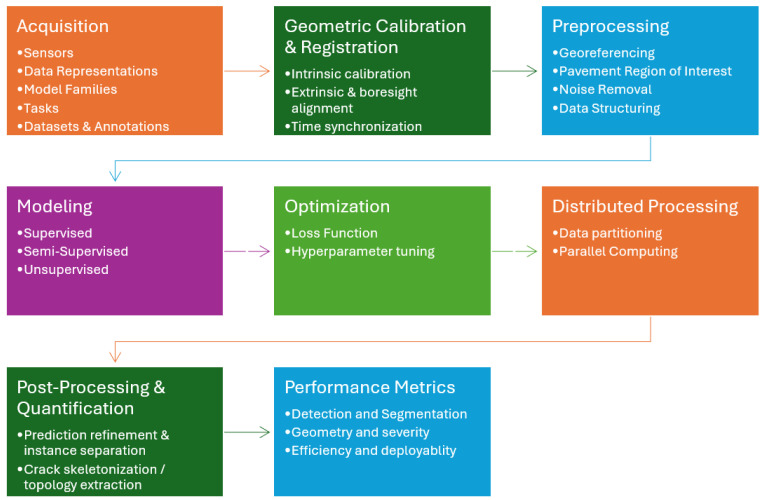
End-to-end LiDAR pipeline for road surface damage classification.

A substantial body of surveys covers road-distress sensing and analytics from various angles, including vision-only reviews, LiDAR-focused overviews, instrumentation/roughness summaries, and systems papers. Vision-centric surveys emphasize CNN/Transformer pipelines on RGB or RGB-D images with brief notes on sensing and field deployment [3,14,23,24,39]. LiDAR-specific reviews focus on acquisition platforms, point cloud preprocessing, and 3D road modeling, with limited attention paid to runtime and distributed execution [40,41,42]. Instrumentation and roughness reviews catalog devices, profilers, and protocols rather than learning pipelines [38,43]. Broader summaries range from image-based and non-image modalities to multisensor and IoT road-condition monitoring systems [43,44]. Table 2 positions these surveys against the eight stages of the 3D pipeline used in this article and shown in Figure 1: data acquisition, geometric calibration and registration, preprocessing, modeling, model optimization, distributed processing, post-processing and quantification, and performance metrics.

Compared with prior overviews, this survey adopts a LiDAR-centered perspective and examines the processing pipeline from acquisition and preprocessing to learning architectures, deployment considerations, and severity-oriented evaluation. It addresses MLS/TLS acquisition and timing, intensity normalization and detrending, native 3D (point/graph/voxel) modeling alongside 2.5D heightmaps, low-label strategies, camera–LiDAR fusion, and, notably, corridor-scale execution tiling/streaming, edge–cloud division, and performance budgeting. It links geometric outputs like crack width/continuity, rut depth, and pothole depth/volume to agency frameworks (e.g., DIM/PCI), a connection that is only briefly treated in most broader surveys [3,14,15,23,38,39,40,42,43,44,45].

**Table 2:** Comparative coverage of survey/review papers across the LiDAR road surface pipeline. Legend: ● well covered, ◗ partially covered, ❍ minimal/absent.

Survey	Acq.	Cal./Reg.	Prep.	Model	Optim.	Distrib.	Post/Quant.	Metrics
[44]	●	◗	◗	●	◗	◗	◗	●
[3]	●	❍	◗	●	◗	❍	◗	●
[14]	◗	❍	●	●	◗	❍	◗	●
[15]	◗	❍	●	●	◗	❍	◗	●
[16]	◗	❍	◗	●	◗	❍	◗	●
[17]	◗	❍	◗	●	◗	❍	◗	◗
[18]	◗	❍	◗	●	◗	❍	◗	●
[19]	◗	❍	◗	●	◗	❍	◗	●
[20]	❍	❍	◗	●	◗	❍	◗	●
[23]	◗	❍	●	●	◗	❍	◗	●
[24]	◗	❍	●	●	◗	❍	◗	●
[42]	●	●	●	◗	❍	◗	●	●
[40]	●	●	●	◗	◗	◗	●	●
[41]	●	◗	●	◗	❍	❍	●	●
[38]	●	◗	◗	◗	❍	❍	◗	●
[43]	●	❍	◗	◗	❍	❍	◗	●
[46]	●	◗	◗	◗	◗	●	●	●
[45]	◗	◗	●	●	◗	◗	●	●
[47]	●	◗	◗	◗	❍	❍	●	◗
[39]	◗	◗	●	●	◗	❍	●	●
This Survey	●	●	●	●	●	●	●	●

Where Table 2 compares prior review papers by end-to-end pipeline coverage, Table 3 complements that view by asking whether each survey explicitly states (i) the target task (e.g., detection, inventory, or severity estimation), (ii) the output expected from that task (e.g., labels, spatial inventory, or dimensional measurements), (iii) the data representation used for analysis (e.g., point clouds, voxels, graphs, or 2.5D rasters), and (iv) the evaluation criteria used to validate those outputs. This distinction matters because studies that report strong segmentation or classification results do not necessarily support inventory-ready products or severity-aware metrology, and surveys that mix these endpoints risk obscuring the practical role of LiDAR geometry in pavement assessment.

Vision-centric surveys [3,14,15,17,18,20,23,24,39] offer strong coverage of detection and segmentation models and dataset practices, with a briefer treatment of acquisition and little coverage of deployment-scale execution. LiDAR-focused articles [40,41,42] provide in-depth discussions of sensing and preprocessing; recent reviews have also expanded the discussion toward road surface inspection workflows and point-cloud processing at the system level [42]. Among the broader systems reviews, [44,46] most consistently discuss multisensor fusion and edge/IoT or operational deployment issues. By design, the present survey spans eight dimensions, adding explicit low-label learning, fusion design, and severity-aware metrology tied to DIM/PCI.

In conclusion, earlier surveys either (i) center on 2D vision with limited LiDAR detail [3,14,23,24,39], (ii) emphasize LiDAR hardware, acquisition, and 3D modeling without sustained attention to full learning pipelines or deployment concerns [40,41,42], (iii) focus on instrumentation or roughness rather than classification analytics [38,43], or (iv) discuss systems at a broader operational level [46]. This survey reviews LiDAR-based pavement analysis across the processing pipeline, including label-efficient methods, deployment considerations, and severity-linked outputs relevant to agency workflows.

### 1.1. Survey Methodology and Scope

This article is a structured review of LiDAR-based methods for pavement-distress detection, inventory generation, and severity estimation. Because survey credibility depends on transparent study selection, the literature corpus was assembled using an explicit multistage search and screening procedure rather than a narrative or convenience-based selection process.

#### 1.1.1. Review Objective and Scope

The review was designed to address four questions: (i) how LiDAR data are represented and processed for pavement-distress analysis; (ii) how prior studies define the target task, particularly Detection, Inventory, and Severity; (iii) which datasets, labels, and evaluation protocols support reproducible comparison; and (iv) what practical constraints affect deployment at corridor scale. The review is LiDAR-centered rather than modality-agnostic: studies were prioritized when LiDAR supplied the primary geometric signal, and multimodal papers were included only when fusion with LiDAR materially affected pavement-distress analysis.

#### 1.1.2. Information Sources, Search Window, and Execution Date

A structured literature search was conducted to identify studies on LiDAR-based pavement distress detection and road surface damage classification. The search covered four major engineering and sensing databases: IEEE Xplore, ScienceDirect, SpringerLink, and MDPI. Searches were limited to publications from 2015 to late 2025, a period chosen to capture the emergence of modern mobile LiDAR acquisition, point-cloud learning, and multimodal pavement-assessment pipelines. All database searches were executed in December 2025.

The search was limited to English-language publications. Journal articles, review articles, and conference papers were included where supported by the database interface and indexing rules of each platform.

#### 1.1.3. Search Strategy

Search queries combined three concept groups: (i) sensing modality, (ii) pavement-distress target, and (iii) analytical task. In practice, the search logic centered on the terms LiDAR, pavement or road surface, defect terms such as crack, rut, pothole, and distress, and task terms such as detection. Searches were performed in title, abstract, and keyword fields where supported by the database interface; where field-restricted searching was unavailable or inconsistent, the closest equivalent full-record search option was used. Database-specific syntax varied across platforms, including the treatment of quotation marks, Boolean grouping, and field filters, but the same conceptual search structure was preserved across all sources.

Table 4 summarizes the principal search configuration used in each database.

The database search returned a total of 719 records, including 49 from IEEE Xplore, 512 from ScienceDirect, 142 from SpringerLink, and 16 from MDPI. To reduce sensitivity to terminology variation, backward and forward citation tracing was also applied during screening to identify relevant studies that might not have been captured by the initial keyword search.

#### 1.1.4. Duplicate Handling and Screening Workflow

Duplicate records were identified using title, DOI, and author-year matching, followed by manual verification where metadata were inconsistent across databases. After duplicate removal, 585 unique records remained for screening.

Title and abstract screening excluded 465 records that were outside the scope of LiDAR-based pavement distress analysis. Common exclusion reasons included non-pavement infrastructure monitoring, geological or geomorphological applications of LiDAR, general computer vision studies unrelated to road surface damage, and structural inspection of buildings, bridges, or tunnels without direct transferability to pavement analysis.

Following title and abstract screening, 120 articles were retained for full-text assessment. Each full-text article was evaluated against predefined eligibility criteria. After full-text review, 53 articles were excluded because they lacked a LiDAR-centered analytical pipeline, were not directly concerned with pavement-surface distress, provided insufficient methodological detail for comparison, or duplicated a more complete peer-reviewed version. The final qualitative synthesis, therefore, included 67 studies.

#### 1.1.5. Eligibility Criteria

Studies were included when they satisfied all of the following requirements: (i) the work addressed pavement-surface distress analysis rather than general road-scene understanding; (ii) LiDAR point clouds or LiDAR-derived geometric representations formed part of the analytical pipeline; (iii) the study contributed to at least one of the review tasks, distress Detection, asset Inventory, or Severity estimation, or reported deployment-relevant issues tightly coupled to those tasks, such as calibration, georeferencing, runtime, or agency-facing measurement workflows; and (iv) the article provided sufficient methodological detail to support comparison of sensing setup, representation choice, labels, task definition, or evaluation procedure.

#### 1.1.6. Exclusion Criteria

Studies were excluded when they (i) focused exclusively on RGB imagery without LiDAR or LiDAR-derived geometry; (ii) addressed defects outside pavement infrastructure (e.g., buildings, bridges, tunnels, or general structural monitoring) without a transferable pavement-analysis methodology; (iii) treated road-scene perception, lane understanding, or autonomous-driving tasks without distress-specific analysis; (iv) lacked sufficient technical detail for meaningful comparison; or (v) were duplicated across databases, in which case only the most complete peer-reviewed version was retained.

#### 1.1.7. Study Grouping and Synthesis Logic

Included studies were synthesized using an eight-dimension pipeline framework rather than pooled quantitative meta-analysis. Each paper was mapped, where applicable, to the following analytical dimensions: Data acquisition, Geometric calibration and registration, Preprocessing, Modeling, Model optimization, Distributed processing, Post-processing and quantification, and Performance metrics. This organization reflects the end-to-end structure of LiDAR-based pavement analysis, from sensing and geometric alignment through learning, scalable execution, measurement, and evaluation.

Within each dimension, studies were compared according to the specific design choices and reporting details available in the source papers. For example, acquisition-related studies were contrasted by platform and sensing configuration; calibration studies by alignment and georeferencing strategy; preprocessing studies by denoising, gridding, detrending, and representation formation; modeling studies by architecture and task formulation; optimization studies by label efficiency, augmentation, loss design, and transfer strategy; distributed processing studies by tiling, streaming, and corridor-scale execution assumptions; post-processing studies by defect aggregation, geometric measurement, and standards-linked quantification; and performance studies by benchmark design, metric selection, and deployment-oriented reporting. Papers spanning multiple dimensions were discussed according to their principal methodological contribution and cross-referenced in other relevant sections when needed.

A formal statistical meta-analysis was not attempted because the literature remains highly heterogeneous in sensor class, calibration practice, spatial resolution, representation, annotation protocol, defect taxonomy, and evaluation criteria, which limits direct quantitative comparability across studies.

#### 1.1.8. Quality and Reporting Assessment

For each included study, the review extracted the following comparison fields whenever available: sensor type and acquisition mode, spatial sampling characteristics, representation used for learning or measurement, defect categories, label granularity, dataset accessibility, evaluation metrics, and any reporting related to calibration, severity measurement, or runtime. This assessment was used to identify not only methodological trends but also gaps in reproducibility, benchmark comparability, and external validity.

#### 1.1.9. Limitations of the Review Protocol

The review protocol has several limitations. First, the search was restricted to major indexed scientific publisher platforms and may underrepresent technical reports, theses, or industry studies. Second, terminology in this field is inconsistent; relevant papers may refer to road damage, surface distress, pavement condition, or specific defect categories without using uniform vocabulary, which may reduce retrieval completeness. Third, the public benchmark ecosystem for LiDAR-based pavement analysis remains limited, so the available evidence is shaped by a relatively small number of datasets, sensing setups, and regions. For these reasons, the present review should be interpreted as a structured and transparent synthesis of the accessible peer-reviewed literature rather than as an exhaustive census of all unpublished or proprietary work.

### 1.2. Survey Organization

Following the task taxonomy introduced above, the remainder of this review is organized around Detection, Inventory, and Severity, with later sections addressing acquisition, datasets, preprocessing, deployment, and evaluation in support of those tasks.

The remainder of the paper is organized as follows. Section 2 reviews data acquisition platforms and annotation considerations. Section 3 summarizes publicly available datasets and discusses dataset quality, comparability, and reproducibility issues. Section 4 examines preprocessing pipelines and LiDAR data representations. Section 5 reviews learning methods organized by task. Section 6 discusses runtime constraints, scalable processing, and deployment considerations. Section 7 summarizes evaluation practice and agency-oriented interpretation of severity measurements. Section 8 then outlines current challenges and research directions. Finally, Section 9 concludes the survey.

## 2. Data Acquisition

Reliable road surface analysis generally depends on disciplined acquisition. For many surface-level road surveys, mobile laser scanning (MLS) is the most commonly used platform.

Airborne and UAV-mounted LiDAR systems extend coverage and reach into constrained areas but generally trade off the spatial detail required to detect small-scale pavement distress. Field studies consistently report that maintaining steady acquisition speed and synchronizing LiDAR, cameras, and IMU/GNSS sensors to a shared clock improve spatial consistency and temporal alignment in mobile scanning systems. The literature emphasizes maintaining stable sensor extrinsics along the route to reduce drift and keep every return anchored to real-world coordinates [2,13,19,22,43].

Among platforms, MLS is the most commonly reported platform for pavement-scale work, TLS supports localized high-precision studies, and airborne laser scanning (ALS)/ uncrewed aerial vehicle laser scanning (UAV-LS) prioritize area coverage over fine detail. When imagery accompanies LiDAR, it adds texture cues and enables fusion, but the literature often suggests that reliability depends at least as much on calibration and timing as on the exact sensor mix [2,9,13,21,22,26,37].

### Annotation and Labeling Considerations

Labels follow the task taxonomy. Detection relies on masks that say where distress exists; Inventory adds instance IDs and types; Severity anchors measurements to standards and ground truth. Keeping these roles separate helps avoid conflating binary detection with grading and makes results plug into DIM/PCI and IRI workflows.

For Detection, annotators paint pixel masks on 2.5D height grids or range images, or assign labels directly to 3D points. The choice tracks the representation: rasters are fast to draw; native 3D preserves geometry for later measurement. Evaluation practices for these tasks are summarized in Section 7.

For Inventory, each defect receives its own outline or mask, along with a type tag (e.g., crack, pothole, rut, patch). Quality is checked with AP/mAP at IoU thresholds and per-instance Precision/Recall to catch splits/merges.

For Severity, labels move from “where” to “how much”. Ground truth includes depths, widths, and volumes from meshes or reference profiles, along with severity grades or indices. Roughness adds longitudinal profiles and IRI against a certified reference. Reconstruction tasks compare the recovered surface to ground truth with RMSE/MAE and completeness (“% within tolerance”). These outputs can then be related to DIM and PCI frameworks.

## 3. Datasets, Benchmark Quality, and Reproducibility

The benchmark ecosystem for LiDAR-based pavement-distress analysis remains limited and fragmented. In contrast to image-based road-damage research, which benefits from several large public benchmarks, LiDAR-centered studies are distributed across a small number of public datasets, partially accessible collections, reconstruction-oriented resources, and many private or project-specific datasets that are not released in reproducible form. This scarcity has direct consequences for cross-study comparison, task coverage, and the validity of severity-oriented claims.

To clarify the current landscape, this review distinguishes among five dataset categories: (i) public LiDAR-native pavement datasets, (ii) partially accessible or limited-release LiDAR datasets, (iii) reconstructed-geometry datasets used for severity or 3D surface analysis but not acquired as native LiDAR benchmarks, (iv) private or study-specific datasets described in the literature without public release, and (v) auxiliary RGB or multimodal datasets used primarily for transfer learning, pretraining, or baseline comparison. Table 5, Table 6, Table 7 and Table 8 summarize these resources and their implications for benchmark validity.

Among the resources identified in the literature, only a very small subset functions as public or semi-public benchmarks for geometry-aware pavement analysis. Table 6, therefore, should not be read as a complete census of all datasets used in published studies, but, rather, as a summary of the principal public or partially accessible datasets that are sufficiently documented to support benchmark-oriented discussion.

### 3.1. Auxiliary RGB and Transfer Benchmarks

Compared with general-purpose urban-scene datasets, road surface distress datasets typically exhibit pronounced class imbalance and a higher proportion of elongated, thin structures (e.g., hairline cracks and sealed joints) embedded in a largely planar background. These characteristics increase the difficulty of semantic segmentation and defect extraction, particularly with respect to geometric continuity, boundary accuracy, and small-object preservation. Therefore, dataset selection has a direct impact on the evaluation of robustness, cross-domain generalization, and the structural fidelity of reconstructed defects.

Collectively, the core LiDAR datasets summarized in Table 6 and Table 7 form a small but growing foundation for geometry-aware pavement-distress benchmarks, and recent primary studies continue to expand the practical evidence base for low-cost pothole quantification, terrestrial LiDAR distress evaluation, and mobile LiDAR documentation workflows [37,49]. Auxiliary RGB and multimodal datasets (Table 8) remain useful for transfer learning and baseline comparison but cannot support depth-based severity validation.

The current landscape of LiDAR pavement datasets remains fragmented across tasks, platforms, and severity definitions, limiting direct numerical comparison and motivating standardized reporting frameworks.

### 3.2. Private and Study-Specific Datasets

A substantial portion of LiDAR-based pavement-distress research is conducted on private, institution-specific, or project-specific datasets that are not publicly released [7,13,36,37,41,49]. These datasets often support important methodological contributions, including rut-depth estimation, pothole metrology, terrestrial crack analysis, or corridor-scale mobile LiDAR workflows, but they do not function as reusable public benchmarks.

This reliance on non-public datasets has three consequences. First, it limits reproducibility because external researchers cannot verify preprocessing, label definitions, detrending assumptions, or severity computations. Second, it weakens cross-paper comparability because reported performance may reflect dataset-specific acquisition density, road type, or defect taxonomy rather than model capability alone. Third, it slows benchmark maturation, especially for severity estimation, where reproducible reference surfaces, tolerances, and measurement protocols are essential.

For these reasons, the present review distinguishes between evidence from private datasets and benchmark-ready public resources. Both are useful for understanding methodological trends, but only the latter can support standardized comparative evaluation.

These limitations reinforce that benchmark availability, not only model design, constrains reproducibility and cross-study comparison in LiDAR-based pavement analysis.

### 3.3. Sensor Characteristics and Dataset Quality Considerations

Although public datasets enable comparative evaluation, their sensing hardware and acquisition protocols differ enough that reported accuracy is not directly comparable across datasets without additional context [40,41]. In LiDAR-based pavement analysis, the limiting factors are often not only model capacity but also geometric fidelity: shallow depth residuals, thin cracks, and small spalls can fall below the effective resolution of the sensing configuration, particularly under motion and at oblique incidence angles [41,54].

**Range precision and depth reliability** 

Severity-related tasks (e.g., rut depth and pothole volume) depend on stable millimetric-to-centimetric depth residuals after detrending and reference-surface fitting [7,13,41]. In practice, the achievable depth reliability depends on the sensor’s ranging noise, scan geometry, and trajectory/georeferencing quality in mobile systems [54,55,56].

**Angular resolution, point spacing, and crack detectability.** 

Thin defects are vulnerable to undersampling: when the effective ground point spacing (across-track or along-track) exceeds the characteristic crack width, detection performance can degrade and continuity metrics can fragment even when overall IoU appears strong [2,6,8]. Mobile systems further exhibit anisotropic sampling, in which point density varies by direction (e.g., denser along scan lines and sparser across track due to vehicle motion) [41,54].

**Scan pattern and incidence angle** 

Rotating multibeam sensors generate non-uniform footprints on the road surface, and shallow incidence angles can reduce height contrast for subtle defects, affecting both detection confidence and measured severity [41,54]. For this reason, dataset descriptions should report mounting height, scan geometry, and acquisition speed whenever possible [40,41].

**Intensity availability and comparability** 

Several pipelines use intensity as an auxiliary cue for cracks and patches. However, intensity is not directly comparable across sensors due to wavelength, automatic gain control, and proprietary calibration, and some datasets omit calibrated intensity entirely [40,41]. When intensity is provided, dataset documentation should clarify whether it is raw, normalized, or radiometrically calibrated [40].

**Platform motion and georeferencing quality** 

Mobile laser scanning (MLS) point clouds inherit errors from GNSS/IMU integration and boresight calibration; these errors propagate into the reconstructed surface and can appear as low-frequency undulations that complicate detrending and bias depth-based severity measures [54,55,56]. Corridor-scale datasets should, therefore, specify the trajectory processing approach (e.g., multipass adjustment, control targets, tightly-coupled estimation) and report expected absolute/relative accuracy [55,56].

**Ground truth modality and measurement validity** 

Label format matters for what can be claimed. Projection-based 2D masks derived from 3D geometry are useful for detection benchmarking but may be insufficient for volumetric severity without explicit 3D reference surfaces or field-measured quantities [7,13,36]. For roughness-related outcomes, longitudinal profile standards (and profiler practices) provide established guidance on measurement and processing assumptions [38,57].

**Recommended minimum metadata for dataset reporting** 

To improve reproducibility and interpretability, dataset releases should report at minimum: (i) sensor model/type and scan pattern, (ii) nominal ranging accuracy and angular resolution, (iii) average ground point spacing (longitudinal and lateral) under the stated speed and mounting height, (iv) availability and calibration status of intensity, (v) georeferencing method and expected trajectory accuracy, and (vi) ground truth generation procedure and its geometric validity for severity tasks [40,41,55,56]. The extent to which current public datasets satisfy these reporting recommendations varies substantially, as summarized in Table 9.

### 3.4. Comparability and Reproducibility Across Datasets

Reported performance across pavement datasets is not directly comparable unless acquisition, labeling, and evaluation protocols are aligned. Differences in platform, sampling density, label definition, and split strategy frequently explain performance variation more than architectural differences.

**Label taxonomy and granularity.** 

Detection datasets vary in whether they provide binary distress masks, multiclass defect categories (e.g., crack types, potholes, patches), or geometry-linked labels for severity [34,35,50,51]. Crack datasets differ in whether hairline cracks, sealed joints, and block cracking are annotated as distinct classes or merged into a single category. These differences affect both IoU/mIoU and instance-level metrics, and should be explicitly acknowledged when comparing results.

**Severity ground-truth definition.** 

Severity datasets differ substantially in how depth, width, or volume are defined and annotated. Some provide continuous geometric ground truth derived from 3D meshes, fitted surfaces, or reference profiles (i.e., regression severity) [7,13,58], whereas others provide ordered categorical labels aligned with inspection manuals (i.e., ordinal severity) [25,26].

**Acquisition platform and density regime.** 

Mobile laser scanning (MLS), handheld LiDAR, stereo/RGB-D, and airborne systems operate under different point-spacing and incidence-angle regimes [40,41,54]. Thin crack detectability is directly tied to effective ground spacing; therefore, reported recall and continuity metrics should be interpreted relative to platform characteristics rather than treated as architecture-only effects.

**Split strategy and leakage risk.** 

Corridor datasets are especially vulnerable to unintended overlap between training and testing partitions if adjacent segments appear in both sets. Geographically disjoint splits are preferable for evaluating generalization across segments or survey runs [3,18]. Studies should state whether splits are random, tile-based, or geographically separated, and whether seasonal or cross-region testing was performed.

**Reproducibility artifacts.** 

Comparability improves when datasets provide official splits, evaluation scripts, and clear severity-mapping procedures. For LiDAR-based severity tasks, publication of detrending parameters, reference surface definitions, and measurement tolerances is necessary for reproducibility [30,31,57]. Without these artifacts, reported geometric accuracy may not be replicable.

**Task suitability summary.** 

Not all datasets support all tasks. RGB-only datasets (e.g., RDD variants) primarily support Detection and Inventory benchmarking but cannot validate depth-based Severity [50,51]. Geometry-rich datasets (e.g., MLS or 3D reconstruction sets) enable Severity evaluation but may be geographically narrow [34,36,41]. Survey comparisons should, therefore, state explicitly which task each dataset validly supports (Detection, Inventory, Severity) rather than implying universal applicability.

### 3.5. Dataset Task Suitability and Comparative Validity

Datasets differ in the tasks they validly support, particularly across detection, inventory, and severity. Table 10 summarizes this distinction for representative datasets reviewed in this survey.

Detection-focused datasets are the most common. RGB-based resources such as the RDD variants primarily support binary or multiclass distress detection and instance inventory benchmarking but do not provide geometric ground truth for depth or volume estimation. These datasets are, therefore, appropriate for evaluating segmentation or classification architectures but cannot support metric severity claims or DIM/PCI-aligned measurement validation.

In contrast, LiDAR-based datasets and reconstruction-oriented datasets provide geometric surface information that enables regression-based severity evaluation. Datasets containing explicit surface geometry or independent measurement references can support quantitative evaluation of crack width, rut depth, or pothole volume. However, many of these resources remain geographically narrow or platform-specific, which limits their usefulness for cross-sensor generalization studies. Datasets collected using a single acquisition platform or sensor configuration are typically unsuitable for evaluating robustness across MLS, handheld LiDAR, or reconstruction-based pipelines.

Only a subset of datasets provide information suitable for agency-oriented severity reporting. PCI or DIM-aligned evaluation requires not only detection labels but also metrically calibrated geometric reference data and clearly defined detrending or reference-surface procedures. Datasets lacking these elements can support detection or inventory benchmarking but should not be used to justify PCI/DIM-linked severity claims.

Thus, comparisons across studies must be interpreted relative to dataset capability rather than model architecture alone. Detection metrics reported on RGB datasets are not directly comparable with severity regression results reported on LiDAR datasets, and cross-platform generalization claims require datasets that include consistent acquisition metadata and sensor characterization.

### 3.6. Severity Dataset Rigor and Metrological Validity

Severity evaluation in LiDAR-based pavement analysis depends not only on segmentation quality but on the geometric validity of the reference data. Unlike detection datasets, severity datasets must support measurement traceability, repeatability, and unit consistency.

**Ground-truth acquisition modality.** 

Severity ground truth is obtained using several modalities: (i) independent 3D meshes or higher-precision scanners [36,41], (ii) profiler-derived reference profiles for rutting or roughness [38,57], or (iii) manual categorical inspection aligned with DIM/PCI standards [30,31]. These modalities are not equivalent. Mesh- or profiler-derived references support regression error analysis (MAE/RMSE), whereas manual categorical labels only support ordinal agreement. Comparisons across studies must, therefore, distinguish regression-based severity validation from bin-based validation.

**Reference-surface definition.** 

Depth and volume measurements require explicit detrending and reference surface fitting [7,13,41]. Datasets differ in whether the reference surface is

- Locally fitted per defect;
- Fitted per tile or per corridor segment;
- Derived from independent reference runs.

If the reference definition is undocumented, reported depth accuracy may reflect fitting assumptions rather than true geometric agreement.

**Effective spatial resolution and sampling limits.** 

Severity reliability depends on effective ground spacing relative to defect scale. When point spacing approaches crack width or shallow rut depth, measurement bias is expected [2,6,54]. Datasets rarely report the minimum detectable defect scale implied by their sampling density. Surveys should interpret reported width/depth accuracy relative to the acquisition regime rather than treating it as model-only performance.

**Unit declaration and calibration.** 

Severity datasets should declare

- Unit calibration (mm, m);
- Pixel-to-metric conversion (for 2.5D grids);
- Profile spacing for rut/IRI computation;
- Volume integration method and sampling step.

In several public datasets, these calibration parameters are not fully specified, limiting reproducibility [34,40,41].

**Repeatability and uncertainty reporting.** 

Profiler standards for roughness specify repeatability and tolerance bands [57]. Comparable reporting is uncommon in LiDAR-based distress datasets. Severity datasets rarely provide repeated acquisitions of the same segment to quantify variance, and confidence intervals for depth or width are seldom published. Without repeatability analysis, severity regression error cannot be fully interpreted.

**Threshold sensitivity in bin-based severity.** 

Datasets aligned with DIM/PCI use discrete thresholds to assign severity levels [30,31]. When measurements lie near threshold boundaries, small geometric deviations can change assigned severity categories. Few datasets provide sensitivity analysis or margin-to-threshold statistics, which are necessary for interpreting disagreement rates in ordinal severity evaluation.

**Implications for survey comparison.** 

For rigorous comparison of severity methods, studies should report

- Measurement error stratified by defect magnitude;
- Tolerance-band compliance (e.g., % within ±3 mm);
- Bias and dispersion separately;
- Threshold-margin analysis for bin assignments;
- Whether evaluation is against geometric reference or manual inspection.

Absent these details, severity claims should be interpreted cautiously, particularly when comparing across acquisition platforms or datasets [7,13,41,57].

In the datasets reviewed here, regression-capable severity validation is primarily supported where independent geometric reference or profile data are available [7,13,36]. In contrast, datasets aligned with DIM/PCI provide categorical severity without explicit measurement uncertainty [30,31]. Few publicly available LiDAR pavement datasets publish repeated acquisitions to quantify repeatability, and reference-surface fitting assumptions are often underreported. As a result, cross-dataset comparison of depth or volume error should be interpreted cautiously.

## 4. Preprocessing and Representation

Several geometric operations (e.g., detrending, reference-surface fitting, and residual computation) recur throughout LiDAR-based pavement analysis and are introduced in context where they are applied. Typical preprocessing pipelines begin by synchronizing clocks and frames, expressing returns in a stable world reference, and partitioning the data into tiles for scalable processing. Point clouds are typically transformed into a projected coordinate system (e.g., UTM, EPSG:32605 [59]) to enable consistent metric analysis and tiling. Field reports repeatedly indicate that disciplined timing and alignment are major determinants of reliability, often outweighing differences in sensor mix [2,9,13,21,22,26,43].

**Figure 2:**
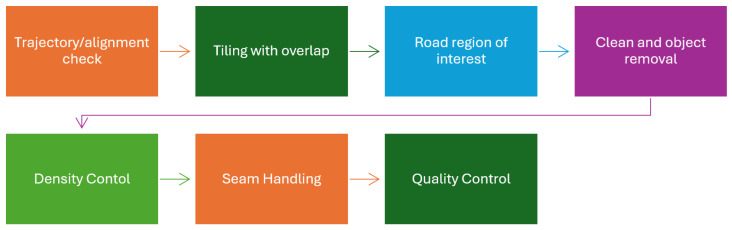
LiDAR preprocessing pipeline.

As shown in Figure 2, preprocessing converts raw LiDAR point clouds into analysis-ready road data by first aligning timestamps and coordinate frames, then partitioning the corridor into short along-road tiles with 10–20% overlap so that cracks and ruts are not clipped at tile boundaries. After tiling, pipelines typically retain the drivable surface, apply ground filters and curb/lane masks or a light semantic pass to close small holes, remove noise such as dust and speckle with simple outlier rules, and exclude cars, rails, poles, and stray vegetation using height and connected-component checks. LiDAR intensity is often normalized with range and incidence-angle compensation so that runs from different days and rigs agree, edges may be padded (mirrored or small overlaps) to avoid boundary artifacts, sampling is commonly equalized with coarse voxel downsampling for steady density, and pipelines often conclude with a quick quality control sweep, basic stats and thumbnails, to catch bad runs early.

### 4.1. Data Representations

Representation limits what the model can “see” and how much geometry survives into measurement. In the literature, representation choice is typically matched to the target task and required severity outputs. As shown in Table 11, three working options recur in pavement work, each with clear trade-offs [5,8,10,11,14].

#### 4.1.1. 2.5D Rasters

**Figure 3:**
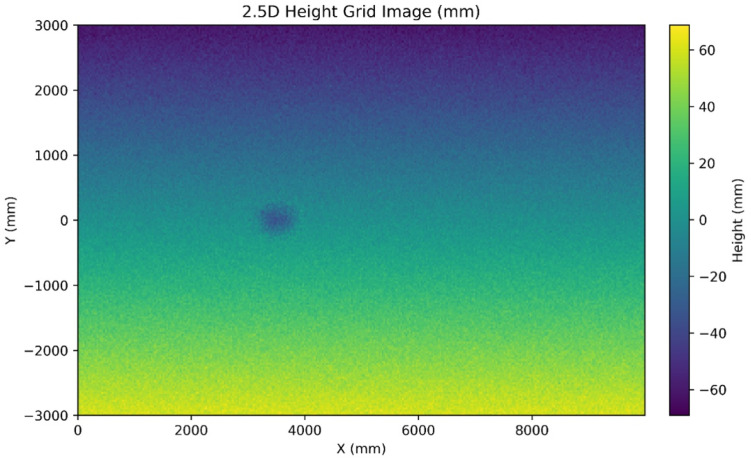
2.5D height grid representation of 30 mm pothole.

Because common models like U-Net operate on 2D (i.e., 2.5D) grids, the raw LiDAR point cloud must be transformed into this structured format. This representation aggregates points into geo-aligned grids (e.g., height or height residual, slope, roughness, intensity, and normals as channels), where height residual denotes the vertical deviation of each point relative to a locally fitted reference surface. Figure 3 demonstrates 2.5D height grids defined over along-road and cross-road coordinates to rasterize elevation or time-aligned depth, which enables the reuse of mature 2D CNN toolchains and efficient training. However, some geometric fidelity relevant to severity measurement is lost [5,10].

#### 4.1.2. Range Images

**Figure 4:**
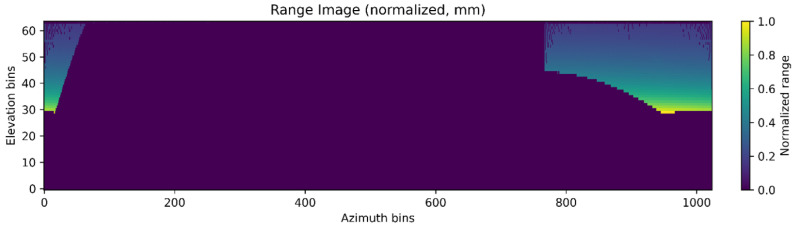
Range image representation of a 30 mm pothole.

The range image representation unwraps the scan to a panoramic image with per-bin features (range, remission, multireturn stats, local curvature). Range images depicted in Figure 4 organize returns in the sensor’s angular frame and are a practical choice for streaming and real-time passes when ultra-fine detail is not the bottleneck [14]. These trade-offs are evident in the contrast between rasterized heightmap approaches, which enable efficient image-based processing, and point- or graph-based methods, which preserve richer geometric detail at higher computational cost.

#### 4.1.3. Native 3D

**Figure 5:**
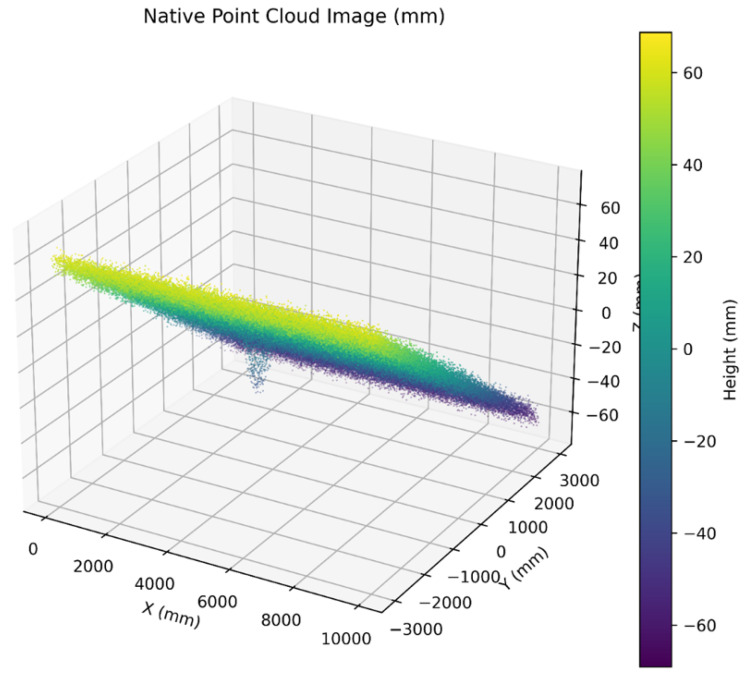
Native point cloud representation of 30 mm pothole.

Native 3D representations retain metric 3D structure and typically use kNN/radius graphs or sparse voxels. Native 3D point sets/graphs shown in Figure 5 maintain depth continuity and neighborhood structure, which is particularly valuable for crack-width and rut-depth estimation, but they require more computing and denser, harder-to-obtain labels [8,11,32].

### 4.2. Task-Dependent Architectural Limitations

Architectural comparisons in the literature often emphasize aggregate metrics (e.g., IoU, F1-score) without clarifying task suitability. For pavement applications, the distinction between Detection, Inventory, and Severity is critical because architectural assumptions affect geometric fidelity, thin-structure preservation, and depth reliability.

Projection-based networks (e.g., U-Net applied to 2.5D grids) inherit discretization error from rasterization. If grid resolution exceeds characteristic crack width, thin defects may be underrepresented or fragmented, even when segmentation metrics appear competitive [60,61]. Additionally, severity tasks requiring volumetric or depth-based measurement depend on stable reference surface estimation; projection smoothing may bias depth residuals [7,13].

Range image representations are efficient but sensitive to sensor-specific angular sampling. Differences in vertical channel spacing or scan pattern can introduce domain shift across datasets [62,63]. As a result, models trained on one sensor configuration may generalize poorly to another without recalibration.

Point-based architectures preserve geometric precision and avoid voxel quantization [64], but local point grouping (e.g., KNN or radius-based selection) can oversmooth thin cracks if the grouping radius is too large relative to the crack width. Performance may also degrade under strong density variation typical of mobile laser scanning [54].

Sparse voxel networks improve computational efficiency and scale to corridor-length datasets [65,66]. However, voxel quantization introduces spatial discretization error. If voxel size approaches or exceeds crack width, geometric detail is suppressed. This limitation is particularly consequential for severity tasks involving width or depth measurement.

Hybrid pipelines mitigate some of these constraints by combining coarse voxel-based detection with point-level refinement. However, they introduce additional design complexity and may propagate early-stage detection errors into severity estimation.

Table 12 summarizes these limitations in task-specific terms to clarify architectural suitability.

Road surface point clouds differ structurally from generic 3D object datasets such as ModelNet or ShapeNet [69,70]. Pavement scans acquired using mobile laser scanning (MLS) systems exhibit strong anisotropy in sampling density, with denser returns along scan lines and sparser coverage across track at operational speeds [54]. The underlying geometry is largely planar with localized depth deviations corresponding to cracks, ruts, and potholes. In addition, defect classes occupy a small fraction of the total surface area, leading to extreme class imbalance in segmentation tasks [71].

Architectural design must, therefore, account for (i) non-uniform sampling density, (ii) thin and elongated structures, and (iii) the need for millimetric geometric fidelity in severity estimation [7,13,41]. Models developed for general object recognition require adaptation to preserve subtle surface discontinuities and depth residuals characteristic of pavement distress.

### 4.3. Architectural Families: Survey-Level Summary

LiDAR-based pavement analysis commonly employs three architectural families: projection-based 2.5D CNNs, native point/graph models, and sparse voxel networks [5,8,64,65]. Rather than treating these as interchangeable design choices, the literature shows that their suitability depends primarily on the target task and required geometric fidelity.

Projection-based methods (e.g., height grids or range images) are widely used for detection and large-scale screening due to their computational efficiency and compatibility with mature 2D segmentation frameworks. However, their discretization can limit thin-crack visibility and bias depth-related measurements when resolution is coarse relative to defect scale.

Point- and graph-based methods operate directly on native 3D geometry and are better suited to preserving thin-structure continuity and supporting severity-linked measurements such as crack width and depth. Their primary limitations are computational cost and sensitivity to local point-grouping scale under anisotropic mobile sampling.

Sparse voxel approaches provide a compromise between efficiency and geometric fidelity by discretizing space while enabling scalable 3D convolution. However, voxel size directly controls the trade-off between throughput and the preservation of small or shallow defects.

In this survey, these architectural families are analyzed in task-specific terms. Their implications for Detection, Inventory, and Severity are discussed within the corresponding subsections (Section 5), where representation choice is interpreted relative to output requirements rather than as an architecture-only comparison.

### 4.4. Architectural Implications for Detection, Inventory, and Severity

- **Detection:** For Detection, graph-based or multiscale point architectures can improve crack continuity and mitigate fragmentation under class imbalance [71].
- **Inventory:** For Inventory, stability under density variation is influenced by preprocessing choices such as density normalization and by how local neighborhoods are defined.
- **Severity:** For Severity, tasks requiring volumetric or depth-based metrics tend to favor point-based or hybrid refinement approaches that avoid coarse voxel quantization.

Architectural selection should, therefore, be guided by geometric precision requirements, expected sampling density, and defect scale. Approaches originally developed for generic object classification [64,67] must be adapted to preserve subtle depth variations and elongated discontinuities characteristic of road surface LiDAR data.

## 5. Methods Organized by Task

To keep the review aligned with the task taxonomy introduced in Section 1, this section organizes prior work into Detection, Inventory, and Severity. Representation choices (e.g., 2.5D rasters versus native 3D) and model families are discussed within each task because suitability depends on the target output and evaluation protocol.

### 5.1. Methods for Detection

Detection answers the question “Is there distress here?” and is commonly formulated as binary screening or semantic segmentation over tiles, rasters, or points.

#### 5.1.1. Representations and Model Families for Detection

Detection performance in LiDAR-based pavement analysis is strongly influenced by representation choice, primarily through its effect on thin-structure visibility, neighborhood context, and computational scalability. The representations introduced in Section 4, 2.5D rasters, range images, and native 3D point/voxel forms, are not interchangeable for detection, particularly under the class imbalance and anisotropic sampling typical of mobile laser scanning [54].

For detection tasks, projection-based representations (2.5D height grids and range images) are widely used due to their computational efficiency and compatibility with mature 2D segmentation architectures. However, their discretization imposes a resolution limit: when grid or angular spacing approaches crack width, thin defects may be undersampled or fragmented even when global IoU or F1 remains competitive [60,61]. As a result, detection performance on elongated structures is often better reflected by recall and continuity-sensitive measures than by overlap metrics alone.

Native 3D point- and graph-based models preserve geometric detail and enable information propagation along crack structures, which can improve continuity under sufficient sampling density [64]. However, detection performance in these models depends strongly on local point-grouping scale (e.g., KNN or radius selection): if the grouping radius exceeds the characteristic crack width, defect signals may be oversmoothed by surrounding pavement points. In addition, computational cost and memory usage increase with point density, limiting throughput for corridor-scale inference [54].

Sparse voxel methods provide a scalable alternative by discretizing space while enabling efficient convolution [65,66]. For detection, voxel resolution directly controls defect visibility: coarse voxels suppress thin cracks, while finer voxels increase computational load. As with rasterization, this introduces a trade-off between throughput and the preservation of small or shallow defects.

Given these representation-dependent effects, detection results should be interpreted in the context of spatial resolution, point density, and grouping scale. Reporting should, therefore, include grid or voxel size, effective ground spacing, tiling strategy, and neighborhood parameters, as these factors often determine thin-structure recall and fragmentation behavior independently of model architecture [54].

#### 5.1.2. Imbalance-Aware Objectives for Thin-Structure Detection

Thin pavement defects (e.g., cracks, sealed cracks, joints, small spalls) occupy a very small fraction of surface points or cells, so dense prediction is dominated by the intact background. Under this imbalance, standard cross-entropy tends to prioritize easy negatives and can suppress recall on thin structures, producing fragmented crack masks even when overall accuracy appears high [71,72,73]. For detection, loss design and sampling policy should, therefore, be treated as first-order determinants of mask completeness and continuity.

**Focal loss.** 

Focal loss reduces the influence of easy negatives by down-weighting well-classified samples and concentrating gradient mass on hard examples [71]:

(1) $$L_{\mathrm{FL}}=-\alpha {\left(1-p_{t}\right)}^{\gamma }\log\left(p_{t}\right),$$

where $p_{t}$ is the predicted probability assigned to the ground-truth class, α balances class weights, and γ controls the degree of down-weighting for easy samples.

**Dice-family losses.** 

Overlap-based objectives reduce sensitivity to class frequency by directly optimizing region agreement [72]. Let $p_{i}\in \left[0,1\right]$ be predicted probabilities and $g_{i}\in \left\{0,1\right\}$ the ground truth. The soft Dice loss is

(2) $$L_{\mathrm{Dice}}=1-\frac{2\sum_{i}p_{i}g_{i}+\epsilon }{\sum_{i}p_{i}+\sum_{i}g_{i}+\epsilon }.$$

Generalized Dice further reweights classes to address extreme imbalance using inverse-volume weights [73]:

(3) $$L_{\mathrm{GDL}}=1-2\frac{\sum_{c}w_{c}\sum_{i}p_{i,c}g_{i,c}}{\sum_{c}w_{c}\sum_{i}\left(p_{i,c}+g_{i,c}\right)},\;w_{c}=\frac{1}{{\left(\sum_{i}g_{i,c}\right)}^{2}+\epsilon }.$$

**Tversky and Focal-Tversky.** 

Detection of thin cracks often requires recall-oriented control of false negatives versus false positives arising from texture, markings, or sensor noise. The Tversky index introduces tunable penalties [74]:

(4) $$\mathrm{TI}=\frac{\sum_{i}p_{i}g_{i}+\epsilon }{\sum_{i}p_{i}g_{i}+\alpha \sum_{i}p_{i}\left(1-g_{i}\right)+\beta \sum_{i}\left(1-p_{i}\right)g_{i}+\epsilon },\;L_{\mathrm{Tv}}=1-\mathrm{TI}.$$

Focal-Tversky concentrates learning on hard cases via an exponent γ [74]:

(5) $$L_{\mathrm{FTv}}={\left(1-\mathrm{TI}\right)}^{\gamma }.$$

**IoU-surrogate losses (Lovász-Softmax).** 

IoU is widely reported for detection masks but is non-differentiable in discrete form. Lovász-Softmax provides a convex surrogate that optimizes an IoU-like objective and is often used for rare classes [75]:

(6) $$L_{\mathrm{Lovasz}}=\frac{1}{\left|C\right|}\sum_{c\in C}\bar{\Delta_{\mathrm{Jaccard}}}\;\left(m^{\left(c\right)}\right),$$

where $\bar{\Delta_{\mathrm{Jaccard}}}$ denotes the Lovász extension of the Jaccard loss over margin errors $m^{\left(c\right)}$ for class *c*.

**Boundary- and topology-aware penalties.** 

Overlap scores can remain stable while thin crack networks fragment. Boundary-focused losses penalize geometric disagreement at class interfaces and can improve edge fidelity [76]. A representative boundary loss uses the distance transform d(·) of the ground-truth boundary:

(7) $$L_{\mathrm{Bnd}}=\sum_{i}p_{i}\;d{\left(g\right)}_{i}.$$

For applications emphasizing connected crack chains, topology-oriented losses such as centerline Dice (clDice) have been used to encourage connectivity during training [77]. These terms are typically combined with Dice and/or cross-entropy rather than used alone.

**Class-balanced reweighting.** 

Class-balanced loss reweights classes using the effective number of samples, reducing dominance of frequent classes while avoiding extreme weights for very rare targets [78]:

(8) $$w_{c}=\frac{1-\beta }{1-\beta^{n_{c}}},\;L_{\mathrm{CB}}=-\sum_{c}w_{c}\;y_{c}\log\left(p_{c}\right),$$

where $n_{c}$ is the number of samples (or pixels/points) in class *c* and β∈[0,1).

**Sampling and mining strategies.** 

Loss design is commonly paired with sampling policies that increase exposure to rare positives. Practical approaches include crack-centric patch sampling, oversampling tiles with non-zero distress coverage, and online hard example mining (OHEM) to focus training on misclassified regions [79]. These strategies should be evaluated using recall- and continuity-sensitive criteria in addition to IoU/F1 when thin-structure completeness is a requirement.

**Detection-oriented practice.** 

A common starting point for highly imbalanced crack detection is a compound objective (e.g., Dice + focal, or Tversky + cross-entropy) paired with a sampling policy that guarantees a minimum fraction of distress-positive tiles per batch. Report loss choice, sampling policy, and any boundary/topology regularizers explicitly, as these choices often determine whether crack masks remain continuous under corridor-scale density variation [54].

Table 13 summarizes common imbalance-aware objectives used for thin-structure detection and their typical effects.

#### 5.1.3. Label-Efficient Detection and Unsupervised Screening

High-quality 3D annotation is labor-intensive for corridor-scale pavement point clouds, particularly for thin cracks and volumetric distress boundaries. For detection, label-efficient and unsupervised screening methods are, therefore, widely used to reduce manual labeling while maintaining acceptable recall on rare distress classes [80,81,82,83,84].

**Self-training and pseudo-labeling for detection.** 

Pseudo-labeling expands a small labeled set by training an initial detector and then using high-confidence predictions on unlabeled tiles as additional supervision. Let $D_{L}$ denote labeled data and $D_{U}$ unlabeled data. A common objective is

(9) $$L=L_{\sup}\left(D_{L}\right)+\lambda L_{\mathrm{pseudo}}\left(D_{U}\right),$$

where λ balances supervised and pseudo-labeled losses [80]. In pavement LiDAR, confidence filtering is typically required to avoid reinforcing early undersegmentation of hairline cracks.

**Consistency regularization under density variation.** 

Consistency methods enforce stable predictions under perturbations such as subsampling, jitter, or rigid transforms. For a transformation τ(·), a representative penalty is

(10) $$L_{\mathrm{cons}}={∥f\left(X\right)-f\left(\tau \left(X\right)\right)∥}^{2},$$

which encourages invariance to acquisition noise and sampling differences [81]. This is relevant for mobile laser scanning, where anisotropic density variation is routine and can otherwise destabilize detection of narrow cracks.

**Self-supervised pretraining for detection.** 

Self-supervised learning reduces label requirements by pretraining representations on unlabeled tiles and then fine-tuning on a small labeled subset. Contrastive formulations are common:

(11) $$L_{\mathrm{contrast}}=-\log\frac{\exp(\mathrm{sim}\left(z_{i},z_{i}^{+}\right)/\tau )}{\sum_{j}\exp\left(\mathrm{sim}\left(z_{i},z_{j}\right)/\tau \right)},$$

where $z_{i}$ and $z_{i}^{+}$ are representations of two augmentations of the same tile, and τ is a temperature parameter [82]. For detection, self-supervised pretraining is generally more effective when augmentations preserve crack-scale geometry rather than heavily downsampling or smoothing thin distress structures, because overly destructive perturbations can suppress the very local cues needed for crack-sensitive representations [8,54,82].

**Cross-modal distillation (when RGB is available).** 

When synchronized imagery accompanies LiDAR acquisition, cross-modal distillation can transfer supervision from an image-based teacher to a LiDAR detector. With teacher soft targets $q_{i}$ and LiDAR predictions $p_{i}$, a typical loss is

(12) $$L_{\mathrm{distill}}=\sum_{i}\mathrm{KL}\left(q_{i}∥p_{i}\right),$$

which can reduce the need for dense 3D labeling [83]. For detection studies, reporting should clarify whether image labels are used directly, distilled, or only used for evaluation.

**Reconstruction-based anomaly detection for screening.** 

Anomaly screening learns a model of intact pavement geometry and flags deviations via reconstruction residuals. Autoencoder objectives commonly minimize

(13) $$L_{\mathrm{recon}}=\sum_{i}{∥x_{i}-{\hat{x}}_{i}∥}^{2},$$

and regions with large residuals are treated as candidate distress [84]. For thin cracks, reconstruction can oversmooth high-frequency discontinuities, reducing anomaly contrast unless resolution is preserved and capacity is controlled.

**Geometry-driven grouping without labels.** 

Geometry-driven grouping identifies connected anomalies directly from local shape cues (valleys, steps, curvature changes) without dense manual masks. Implementations appear in MLS time-grids and range images as region growing under constraints on depth variation, planarity, and orientation continuity, and in native point clouds via clustering or surface-based grouping [5,10]. The output is an interpretable candidate map that can be evaluated as a detector and then passed to inventory or severity stages when geometric measurement is needed [7,13,36].

**Failure modes specific to label-efficient detection.** 

Label-efficient methods introduce characteristic risks. Pseudo-labeling can amplify systematic bias when early predictions miss rare crack pixels [80]. Consistency regularization can suppress legitimate crack-scale variation when perturbations interact with anisotropic sampling [54,81]. Contrastive pretraining can prioritize dominant planar structure and underrepresent sparse distress features without targeted fine-tuning [82]. Reconstruction-based screening can underestimate shallow distress while overresponding to textured but structurally intact regions [84]. Taken together, these failure modes suggest that evaluation should include recall- and continuity-sensitive criteria rather than IoU alone, particularly for thin crack structures [8,10,11,20,32,85].

**Reporting practice for label-efficient detection.** 

For comparability, studies should report (i) labeled fraction and seed-set selection, (ii) pseudo-label thresholds and update schedule, (iii) perturbations used for consistency training, (iv) pretraining corpus size and augmentations, and (v) whether evaluation includes thin-structure continuity measures alongside IoU/F1 [8,10,11,20,32,85].

#### 5.1.4. Fusion for Detection

When imagery accompanies LiDAR acquisition, fusion is most commonly used to improve detection robustness rather than to replace geometric measurement. In pavement settings, LiDAR supplies shape and scale cues that stabilize detection under illumination variation, while RGB contributes texture information that can reduce confusion between distress and visually similar patterns (e.g., stains, markings, joints) [2,3,4,5,6,7,8,9,10,11,12,13,14,86]. Hence, fusion is typically motivated by two detection objectives: (i) reducing false positives by requiring cross-modal agreement, and (ii) improving recall in low-contrast or shadowed scenes where texture cues are weak in one modality. A practical limitation in some RGB–LiDAR acquisition setups is partial field-of-view overlap: the camera may capture only a subset of the pavement area sampled by LiDAR, which can restrict fusion to the region jointly observed by both sensors.

Fusion strategies are commonly categorized as early, mid-level, or late integration [62,87]. Early fusion stacks aligned channels (e.g., RGB with LiDAR-derived height/range grids) into a single encoder and can achieve strong detection performance when alignment is precise [5,86,88,89]. Mid-level fusion maintains modality-specific encoders and merges features via concatenation, attention, or gating, offering increased tolerance to minor calibration error at the cost of additional compute [2,9,13,21,26]. Late fusion combines independent predictions using voting or weighted combination, often providing the most stable behavior under drift but smaller incremental gains than tightly coupled designs [71,90].

For detection of thin cracks, the practical constraint is that calibration and synchronization error can approach the spatial scale of the target. Small misalignment may shift RGB evidence relative to a LiDAR height residual, weakening fusion benefits and, in early fusion, introducing systematic inconsistencies that increase false alarms [91]. For this reason, the detection-related claims of fusion papers are most convincing when calibration/timing quality is reported and single-stream baselines are evaluated under identical splits [2,9,13,21,22,26]. Where possible, sensitivity to synthetic misalignment or clock skew provides a clearer indication of whether reported gains will persist in corridor-scale operation [2,9,13,21,22,26].

Published RGB–LiDAR fusion studies in pavement assessment generally suggest that fusion can improve detection robustness when calibration and synchronization are stable, particularly in scenes where geometric and photometric cues are complementary [2,9,13,21,26]. However, the magnitude of reported gains is difficult to compare across studies because datasets, split strategies, alignment quality, and reporting conventions vary substantially across published experiments. For that reason, this survey treats early-, mid-, and late-fusion differences primarily as a design synthesis rather than as a uniform empirical ranking.

However, the literature indicates that reported fusion benefits are sensitive to alignment quality [2,9,13,21,22,26,91]. When extrinsic calibration error or temporal skew approaches the spatial scale of thin cracks, early-fusion designs may become fragile because the two modalities no longer support the same local evidence [91]. Mid-level and late-fusion strategies are often described as more tolerant to minor misalignment, but this should be interpreted as a qualitative tendency rather than a universally demonstrated ranking across benchmarks. Overall, the literature supports the view that realized fusion benefit depends at least as much on alignment quality and acquisition regime as on fusion taxonomy itself.

Because the available studies do not use a common benchmark with standardized calibration diagnostics, the comparison in Table 14 should be read as a qualitative design-oriented summary rather than as a quantitative meta-analysis.

### 5.2. Methods for Inventory

Inventory produces defect instances, counts, and corridor-level maps (e.g., pothole instances, crack segments, patch regions), typically requiring postprocessing and stitching across overlapping tiles.

#### 5.2.1. Instance Extraction and Duplicate Suppression for Inventory

Inventory requires converting dense detection outputs into discrete defect instances with stable counting and corridor-level localization. In the reviewed literature, this step is often underspecified, even though it can dominate errors in reported instance counts, length totals, and mapped defect locations [3,14,15,24]. This sensitivity arises because pavement defects, particularly cracks, form continuous structures that may span multiple tiles or be fragmented by thresholding, overlap boundaries, or small gaps in detection. As a result, instance counts and lengths can depend as much on postprocessing rules (e.g., segmentation continuity, gap thresholds, and stitching policy) as on the underlying detection model.

**From masks to instances: connected components and clustering.** 

For projection-based pipelines, a common approach is to threshold the predicted probability map and extract instances via connected-component labeling on the 2.5D grid or range image plane. Small components are filtered by area, shape, or minimum depth contrast to reduce spurious detections from texture or sampling artifacts [5,10]. For native 3D pipelines, instances are typically obtained by clustering points predicted as distress (e.g., DBSCAN-style density clustering or region growing under geometric constraints), followed by simple geometric filtering based on size, planarity, and depth residual statistics [7,13,36]. These steps are also used to separate nearby potholes or depressions that would otherwise merge under a single mask.

**Crack instances: skeleton-to-segment construction.** 

Crack inventory is frequently reported as length per unit area, segment counts, or corridor-length summaries. Because cracks are thin and elongated, instance extraction commonly proceeds by skeletonizing the predicted crack mask (on a 2.5D grid) or by constructing a centerline graph from predicted crack points, then splitting the skeleton into segments using branch points, gap thresholds, or maximum segment length constraints [8,10,11,20,32,85]. Crack inventory benefits from explicit segment definitions because the same physical crack may be counted as a single instance or as multiple instances depending on the chosen gap-handling and branch-splitting criteria.

**Overlap tiling and duplicate suppression.** 

Overlap-aware tiling is a common practical strategy for long LiDAR strips because it reduces boundary truncation of elongated defects during patch-based processing. Overlap creates duplicate detections that must be reconciled to prevent double counting and to preserve consistent instance geometry. Common strategies include the following: (i) retaining instances whose centroids fall within a tile’s non-overlap “core” region, (ii) merging overlap instances using IoU on projected masks or distance-based matching of centroids and extents, and (iii) using consensus rules (e.g., union or intersection) to stabilize crack continuity across seams. For crack skeletons, overlap reconciliation can be performed by snapping endpoints within a tolerance and merging segments when the joining angle and gap are consistent with a single polyline [8,11,32].

**Recommended reporting for inventory pipelines.** 

Inventory studies are most comparable when they report the binarization thresholding policy, the instance extraction method (connected components, clustering, or region growing), post-filtering criteria (minimum area, length, or depth residual), crack-segment definition rules, and the duplicate-suppression policy used under overlap tiling [3,14,15,24]. Without these details, AP/mAP and count-based results are difficult to interpret and are not comparable across datasets or processing setups.

#### 5.2.2. Corridor-Level Geo-Stitching and Aggregation

Corridor inventories are typically produced by stitching tile-level outputs into a continuous georeferenced map that supports counts and quantities per management section (e.g., per kilometer or per lane segment). Even when the detection model is fixed, differences in stitching and aggregation can change reported inventory results, particularly for elongated defects that span multiple tiles.

**Georeferenced normalization.** 

Most corridor workflows begin by expressing tiles in a consistent road-aligned frame (longitudinal–lateral–vertical) and associating each tile with a georeferenced extent along the route. This step is essential for stable aggregation across runs and for comparing inventory summaries between corridors or survey dates [40,41,54,55,56].

**Overlap reconciliation.** 

Because tile overlap can reduce boundary truncation, overlap regions should be reconciled before corridor-level summaries are computed. Looking across prior studies, reported approaches fall into three main categories: (i) core-region retention, where only detections whose centroids lie in the interior of each tile are retained; (ii) match-and-merge, where overlap instances are paired using proximity and extent similarity and merged into a single instance; and (iii) consensus fusion, where overlap predictions are combined to stabilize thin structures, for example, by intersecting or averaging probability fields prior to binarization. The chosen policy influences both duplicate suppression and crack continuity across seams.

**Cross-tile continuity for elongated defects.** 

For cracks and rut bands, the principal stitching objective is continuity rather than only duplicate removal. Looking across prior studies, this is typically addressed by linking endpoints or boundaries across adjacent tiles using spatial tolerance criteria and then recomputing connected components or skeleton segments on the stitched corridor strip. When reported, continuity-aware linkage is often evaluated with fragmentation measures rather than solely with tile-level IoU [8,10,11,20,32,85].

**Aggregation to corridor summaries.** 

After reconciliation, inventories are aggregated into map layers and summary statistics (counts, total crack length, distressed area fraction, rut length) over declared spatial units (e.g., per 10 m, per 100 m, per kilometer). For comparability, studies should state the aggregation unit, whether quantities are lane-normalized, and whether inventory metrics are computed before or after stitching [3,14,15,24].

**Reporting considerations.** 

At minimum, corridor inventory reporting should specify the tiling scheme (tile length, overlap), the overlap reconciliation policy, and the spatial unit used for aggregation. When georeferencing quality is a limiting factor, reporting should include trajectory accuracy assumptions and whether realignment or map-matching was applied before inventory computation [40,41,54,55,56].

### 5.3. Methods for Severity Estimation

Severity estimates geometric quantities (width/depth/volume/rut profile) or ordinal severity bins, and maps them to DIM/PCI-style decision categories.

#### 5.3.1. Metrology Pipelines for Severity-Linked Geometry

Severity estimation requires converting detections into quantitative measurements that remain reliable despite roadway slope, lateral cross-fall, and variation in acquisition conditions. In LiDAR-based pavement studies, the reliability of depth-linked quantities is often limited by detrending, reference-surface definition, point spacing, and georeferencing stability rather than by the segmentation backbone alone [7,13,41,54,55,56]. For comparability, severity reporting should state units, sampling step, the detrending procedure, and how the local intact surface was defined for reference fitting [30,31].

**Detrending and coordinate framing.** 

Raw elevation includes global roadway grade, cross-fall, and low-frequency undulations from trajectory error. These components are removed through detrending, which estimates the underlying roadway surface and removes global slope and curvature so that local deviations associated with distress can be analyzed [41,54]. Report whether detrending is performed by fitting a plane, polynomial surface, moving-window regression, or profile-based filtering, and report window sizes or control points when applicable [7,13]. For mobile laser scanning, trajectory/georeferencing errors can introduce long-wavelength bias that propagates into depth residuals and may be mistaken for rutting if not handled explicitly [54,55,56].

**Reference-surface fitting.** 

Depth and volume measurements depend on an estimate of the intact pavement surface surrounding the defect, typically obtained through reference-surface fitting, which defines the baseline against which depth is measured. Common practices fit a local plane or low-order surface using points in a neighborhood outside the detected region, then compute vertical residuals (i.e., height residuals) within the defect mask relative to that reference [7,13,41]. The fitted reference should exclude outliers (e.g., debris, vehicles, curb returns) and should be described with its fitting rule (least-squares, robust regression) and neighborhood selection criteria.

**Crack measurement: width, length, and continuity.** 

For cracks, severity-related quantities typically include total length (m), width statistics (mm), and continuity descriptors (e.g., maximum continuous segment length, gap counts above a stated tolerance) [2,6,30,31]. Width estimation is sensitive to point spacing and grid resolution: when effective ground point spacing approaches crack width, reported widths can be biased low and continuity can fragment even if IoU remains acceptable [2,6,8]. Studies should report the width-estimation method (e.g., mask boundary distance on a 2.5D grid, point-to-centerline distance in 3D) and the spatial calibration used to convert pixels or bins to millimeters [30,31].

**Pothole and depression measurement: depth percentiles and volume.** 

For potholes and depressions, common outputs include area (m^2^), maximum depth $d_{\max}$ (mm), percentile depths (e.g., $d_{95}$), and volume (m^3^) computed from detrended residuals integrated over the defect region [7,13,36,49]. Volume estimates require a declared sampling step (grid cell size or point interpolation scheme) and a stated rule for integrating residuals (e.g., summation over cells or triangulated mesh). Because depth residuals depend on the reference surface, reporting should include the neighborhood definition used for fitting and whether the reference is recomputed for each defect or shared over a tile [7,13,41].

**Rutting: transverse profiles and wheelpath depth.** 

Rutting is typically summarized as rut depth (mm) per wheelpath derived from transverse profiles. A common approach estimates a reference cross-section (e.g., straightedge or fitted cross-fall) and reports the maximum deviation within wheelpath bands, along with rut length and optional rut area [38]. Studies should report profile spacing along the corridor, wheelpath definition, and whether profiles are smoothed or filtered prior to depth extraction, as these choices affect both measured depth and repeatability [38,41].

**Roughness linkage: longitudinal profiles and IRI.** 

Roughness metrics such as the International Roughness Index (IRI, m/km) are derived from longitudinal profiles and a quarter-car model. When IRI is reported, it should be evaluated against a certified reference/profiler and accompanied by absolute and relative error or repeatability statistics [38,57]. Because IRI is sensitive to profile filtering and sampling assumptions, reporting should state the profile extraction method and any preprocessing applied to remove non-pavement artifacts [57].

**Minimum reporting for severity metrology.** 

For severity-linked outputs, a minimally informative report includes (i) units and spatial calibration, (ii) sampling step or effective point spacing, (iii) detrending method and window or fit parameters, (iv) reference-surface fitting procedure and neighborhood definition, and (v) error statistics against an independent ground truth where available [30,31,41,55,56]. Without these details, severity values are difficult to compare across datasets and platforms, even when segmentation scores appear similar [7,13,41].

#### 5.3.2. Uncertainty and Threshold Sensitivity

Severity estimates derived from LiDAR are typically interpreted through thresholded decision rules (e.g., DIM/PCI severity bins), so measurement uncertainty should be treated as part of the severity result rather than as a separate implementation detail [30,31]. Primary uncertainty sources include ranging noise, incidence-angle effects, trajectory/georeferencing error in mobile systems, and reference-surface fitting error after detrending [41,54,55,56]. These effects are amplified near severity thresholds, where small depth or width perturbations can change the assigned category and downstream deduct values [30,31].

The formal PCI/DIM mapping equations and related threshold-sensitivity expressions are presented in Section 7. Here, the emphasis is on reporting requirements for severity-linked methods: (i) declare units and sampling step; (ii) specify detrending and reference surface definition; (iii) report measurement error statistics (bias and dispersion) against an independent reference where available; and (iv) include tolerance-band rates (e.g., percentage within ±3 mm) stratified by severity level [7,13,41,57]. When severity categories or PCI values are reported, studies should also state how uncertainty propagates to class assignment, and should summarize sensitivity around the relevant thresholds rather than relying only on aggregate accuracy [30,31,38].

## 6. Scalable Deployment and Distributed Processing

Distributed processing can make long corridor surveys more tractable by partitioning large LiDAR strips into smaller processing units, often with limited overlap to reduce boundary truncation of elongated defects. In the literature, distributed processing is typically described as a practical approach for handling corridor-scale LiDAR data, with effectiveness depending on the sensing setup, tiling strategy, and compute environment [14,15,38,92].

**Figure 6:**
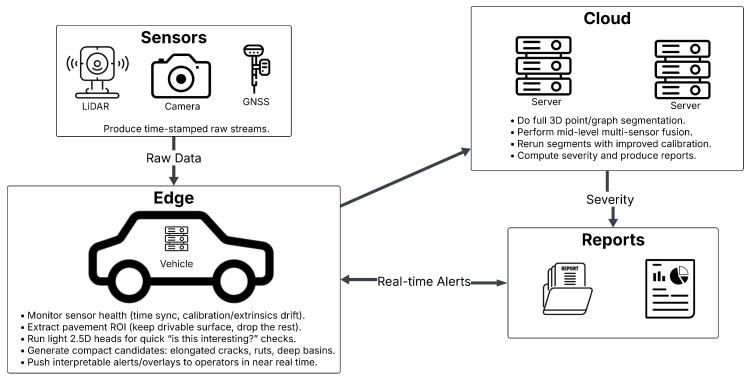
Conceptual edge–cloud workflow for corridor-scale mobile laser scanning (MLS) pavement assessment.

The resulting data volume often exceeds what can be processed directly at the acquisition site (i.e., on the edge device). As summarized in Figure 6, edge–cloud partitioning is commonly described in the literature as a practical strategy for corridor-scale pavement assessment. At the edge, lower-cost steps such as sensor-health monitoring, road region of interest (ROI) extraction, lightweight 2.5D inference for rapid alerts, and candidate generation for elongated cracks, ruts, or deep basins may be performed close to acquisition. These intermediate outputs are comparatively compact and can support near-real-time operator awareness. Cloud-side processing is then used for more computationally intensive stages, including full 3D point/graph segmentation, multisensor fusion, recalibration or reprocessing when needed, and severity measurement and reporting. In the road-damage literature, this partition is best interpreted as a reported design pattern for balancing latency-sensitive screening with heavier back-end analysis rather than as a fixed deployment architecture [15,92].

In deployment-oriented studies, performance budgeting is ideally treated explicitly rather than only retrospectively. Deployment-oriented pipelines often track frames per second, latency, and throughput, alongside accuracy, so that bottlenecks are visible and fixes are targeted. Studies that report both quality and timing are more operationally informative; IoU/F1 or mAP are, therefore, best interpreted alongside tile rate and end-to-end latency; otherwise, results are hard to operationalize [15,92]. Batching and asynchronous queues help absorb rate spikes when traffic, occlusions, or dense urban geometry temporarily increase point counts; reported systems benefit from explicitly defined back-pressure and drop policies to avoid silent failure modes [15,92].

Operational reliability often depends on routine monitoring steps that are easy to overlook. Reported operational frameworks often include continuous time-synchronization checks and extrinsic-calibration monitoring; when drift crosses a threshold, some reported systems fall back to geometry-only candidate generation and tag the segment for cloud reprocessing. Some deployment-oriented studies describe inspection and review stages for checking misalignment, missing lane coverage, or spurious reflections before outputs are incorporated into downstream inventory workflows [43,92].

At fleet scale, hardware variety is the rule rather than the exception. Profiler-style MLS units may support corridor-scale screening, but such claims should be interpreted in light of hardware, density, and throughput assumptions rather than treated as universal deployment facts [38,92]. Lower-density LiDAR configurations may still support selected pothole-screening and sizing workflows, but their usefulness depends on the sampling density, detrending strategy, and robustness of the reference-surface fit used for depth or volume estimation [7,13]. As acquisition setups diversify, scalable processing depends on consistent tiling, interface definitions, and reporting conventions across the pipeline; in this survey, these considerations are better framed as deployment design principles than as settled architectural guarantees [92].

In the deployment-oriented literature, cloud-side post-processing is sometimes used to convert detections into agency-relevant measurements and DIM- or PCI-related categories [15,30,31,38,92]. The same studies often report timing, tile counts, and sensor-quality summaries alongside these outputs to support auditability and operational interpretation [15,30,31,38,92].

### 6.1. Efficiency and Deployability

Across deployment-oriented studies, throughput, end-to-end latency, hardware configuration, and accuracy are among the reporting elements most relevant for interpreting practical scalability. Corridor-scale studies emphasize that accuracy without timing is difficult to operationalize; the literature, therefore, supports reporting both, along with batching and queue-management settings for scenes with elevated processing load [15,46,92].

**Protocols, splits, and robustness** 

For reproducibility and comparison, published studies are most informative when they report sensor platform (MLS/TLS/UAV-LS), point density, speed, and synchronization. Geographically disjoint splits are generally preferable to reduce spatial train–test overlap along continuous corridors. Seasonal and cross-domain splits are less common in LiDAR studies, but related image-based road-damage benchmarks show the value of multiregion evaluation for assessing transfer and robustness [3,18,50,51,52]. New 3D/2.5D datasets (e.g., RSRD-Seg) support more standardized reporting for unevenness segmentation, crack segmentation, and 3D pothole geometry; for such tasks, mIoU is appropriate for segmentation, whereas RMSE, MAE, and volume error are more appropriate for reconstruction-oriented evaluation [35,36].

**Calibration, alignment, and fusion diagnostics** 

Fusion studies are most interpretable when they include RMS reprojection error on checkerboard or scene constraints, point-to-plane residuals on the road surface, and a clock skew estimate. Sensitivity analyses are especially informative when they quantify accuracy loss under synthetic misalignment or desynchronization. Studies that report calibration and timing control generally provide clearer and more interpretable evidence of fusion gains than studies that do not [2,9,13,21,22,26].

A balanced evaluation typically combines detection or segmentation scores to establish where defects are, geometry- and severity-oriented measures to quantify how much they matter within DIM/PCI, and efficiency diagnostics to show whether the method can scale. The cited surveys and studies converge on this balanced bundle; adopting it makes LiDAR-based results directly actionable in pavement management and comparable across platforms and seasons [2,3,6,7,13,14,15,24,30,31,38,40,44].

### 6.2. Runtime Complexity and Latency Trade-Offs in Corridor-Scale Deployment

Corridor-scale LiDAR surveys generate millions of points per kilometer depending on acquisition speed, scan frequency, and sensor configuration [54]. Practical deployment, therefore, depends not only on segmentation accuracy but also on computational complexity, memory footprint, and end-to-end latency.

**Preprocessing and Tiling Complexity.** 

Let *N* denote the number of points within a processing tile. Basic preprocessing operations such as ground extraction and detrending typically require neighborhood search, resulting in complexity of approximately

(14) O(NlogN),

when spatial indexing structures such as k-d trees are used [93]. While linear in practice for fixed neighborhood size, memory locality and I/O bandwidth often dominate runtime for large tiles.

**Projection-Based Models.** 

For 2.5D grids or range images with spatial resolution H×W, convolutional inference scales approximately as

(15) $$O(HWK^{2}C),$$

where *K* is kernel size and *C* is the number of feature channels. Because these tensors are dense and structured, they benefit from optimized GPU kernels and can achieve real-time performance on embedded hardware [62].

However, increasing spatial resolution to preserve crack-level detail increases both memory and latency quadratically with grid dimension.

**Point-Based Networks.** 

Point-based architectures require local point grouping (e.g., KNN or radius-based selection) and feature aggregation. For *N* points and *k* neighbors per point, complexity is approximately

(16) O(NlogN+Nk),

depending on indexing implementation [61]. As point density increases, memory and latency scale proportionally with *N*, limiting throughput in high-resolution MLS data.

**Sparse Voxel Convolution.** 

Sparse convolutional networks operate only on occupied voxels. Let *M* denote the number of active voxels. Inference scales approximately as

(17) $$O(MK^{3}),$$

with memory proportional to *M* rather than full grid volume [65,66]. Finer voxel resolution increases *M*, trading geometric fidelity for computational load.

**Edge Versus Cloud Partitioning.** 

In reported deployment workflows, latency-sensitive screening is often assigned closer to acquisition, while more computationally intensive refinement and reporting are handled offline or in back-end processing environments [15,92]. Within that division, lightweight projection-based or coarse voxel methods are commonly associated with rapid screening, whereas higher-resolution refinement, multistage fusion, and reprocessing of flagged segments are more often discussed in back-end settings [15,92].

Data transmission cost must also be considered. If each point carries *d* features (e.g., x,y,z,I), raw upload scales as

(18) O(Nd),

which can exceed available bandwidth for corridor-scale surveys without compression or tiling [54].

**Latency–Accuracy Trade-off.** 

Resolution reduction (through voxel coarsening or point subsampling) reduces inference time but may suppress thin cracks whose width approaches the effective sampling interval. Empirical LiDAR segmentation studies demonstrate performance degradation on small objects when resolution decreases [61,62]. For pavement applications, this effect directly impacts detection recall and continuity metrics.

**Recommended Reporting Standards.** 

To ensure reproducibility and fair comparison across deployment strategies, studies should report the following:

- Hardware configuration (GPU/CPU model and memory).
- Input tile size and average point density.
- Preprocessing time and inference time separately.
- Peak memory consumption.
- Accuracy under identical spatial resolution.

Without explicit latency and complexity reporting, claims of corridor-scale scalability remain difficult to evaluate.

The literature indicates a recurring trade-off between computational efficiency and geometric fidelity: projection-based methods are often associated with lower-latency screening, point-based methods with higher geometric precision at greater computational cost, and sparse voxel methods with intermediate trade-offs [54,61,62,65,66]. Reported deployment choices, therefore, depend strongly on whether the study prioritizes rapid screening, detailed geometric measurement, or a balance between the two.

## 7. Evaluation Framework and Agency Integration

The objective of LiDAR-based pavement assessment extends beyond segmentation accuracy. Geometric measurements derived from point clouds are ultimately interpreted within agency frameworks such as the Pavement Condition Index (PCI) and the Distress Identification Manual (DIM) [30,31]. These frameworks rely on discrete severity definitions and deduct-value procedures. Consequently, evaluation must consider not only overlap metrics but also geometric reliability and threshold stability.

Table 15 summarizes the principal metric families used in pavement-distress evaluation and their limitations when applied to LiDAR-based measurements.

### 7.1. Severity Definitions and Output Types

Severity assessment can be formulated in three primary ways, which differ in required ground truth, evaluation protocol, and how results connect to agency decision frameworks [30,31,38,57]. The first formulation treats severity as a regression problem, estimating continuous geometric quantities such as crack width, rut depth, pothole depth, or volume. These outputs are naturally expressed in declared units and can be validated against independent measurements using error statistics (e.g., MAE/RMSE and bias), often stratified by distress type and magnitude [7,13,41].

A second formulation treats severity as an ordinal classification problem. Here, measurements or learned features are mapped into ordered categories (e.g., low/medium/high), typically using thresholds on geometric quantities or learned ordinal models. Ordinal bins simplify reporting and can align with inspection manuals, but they introduce threshold sensitivity: small measurement deviations near category boundaries may flip the assigned severity class [30,31]. As a result, ordinal evaluation should state the bin definitions explicitly and report confusion patterns near thresholds, rather than only overall accuracy.

The third formulation is index mapping to agency frameworks such as FHWA LTPP DIM and PCI (ASTM D6433-23), where severity and quantity are combined into standardized categories and deduct values [30,31]. This mapping is discontinuous with respect to measurement error and depends on declared assumptions (units, detrending, reference surface definition, and sampling step). Studies that report PCI- or DIM-linked outcomes should, therefore, make the mapping procedure explicit and provide measurement error context to support interpretability [38,57].

### 7.2. Evaluation by Task

The literature supports evaluating LiDAR-based pavement analysis according to the task being performed rather than treating all reported metrics as interchangeable. For Detection, studies most often report overlap-based measures such as IoU/mIoU and Dice/F1, but crack-focused work also shows the value of recall- and continuity-sensitive reporting because small gaps can reduce structural usefulness even when overlap remains acceptable [8,10,11,20,32,85].

For Inventory, the emphasis shifts from region labeling to instance correctness and corridor-level aggregation. In this setting, AP/mAP, counting error, localization error, and duplicate-suppression behavior are generally more informative than pixel overlap alone, particularly when overlap tiling, stitching, or crack fragmentation affect the final inventory [3,14,15,24].

For Severity, overlap metrics by themselves are not sufficient because the relevant outputs are geometric quantities such as width, depth, volume, rut profile, or roughness. Accordingly, severity-oriented studies are more appropriately interpreted through regression-style error measures such as MAE, RMSE, bias, and tolerance-band compliance, together with threshold-sensitive analysis when results are mapped to agency-facing severity categories [7,13,30,31,36,38,57].

Taken together, these studies suggest that metric choice is task-dependent: detection scores do not by themselves establish severity validity, and instance-level results do not substitute for metrological reliability.

### 7.3. Comparability Limits Across Datasets and Platforms

A recurring limitation in the literature is that reported results are often not directly comparable across studies because acquisition density, split strategy, ground-truth definition, and reference-surface assumptions differ substantially. For thin pavement defects, effective point spacing and raster or voxel resolution directly affect crack visibility and continuity, so similar IoU or F1 values may reflect materially different levels of geometric fidelity under different sensing regimes [2,6,40,41,54].

Instance-level results are, likewise, shaped by post-processing choices, including connected-component rules, crack-segment definitions, overlap reconciliation, and corridor aggregation units [3,14,15,24]. For severity studies, comparability is further constrained by detrending procedure, local reference-surface fitting, unit calibration, and the form of ground truth used for validation, such as profiler data, independent meshes, or manual inspection [7,13,30,31,36,41,57].

Viewed together, these studies suggest that cross-dataset comparison is most informative when sensing conditions, measurement definitions, and evaluation procedures are sufficiently aligned. In that sense, differences in reported scores are most meaningful when the underlying task and metrological assumptions are comparable.

### 7.4. Continuity and Structural Evaluation for Crack Networks

The crack-analysis literature repeatedly shows that overlap metrics alone do not fully capture whether thin crack networks are preserved as connected structures. This matters in pavement assessment because fragmentation can change crack length, instance counts, and severity-linked summaries even when IoU or F1 appears stable [64,94,95,96,97]. For that reason, crack-centric studies often benefit from reporting at least one continuity-aware measure alongside conventional overlap scores.

To make such reporting reproducible, the underlying definitions should be stated explicitly. Let *G* denote the ground-truth crack network and $\hat{G}$ the predicted crack network after skeletonization, with connected components C(G) and $C(\hat{G})$, respectively. Let L(G) denote the total ground-truth crack length and let $L_{\tau }\left(G,\hat{G}\right)$ denote the length of ground-truth crack centerline that is matched by the prediction within a spatial tolerance τ.

A simple break rate can be defined as the excess number of predicted connected crack components relative to the reference network, normalized by the number of reference components:

(19) $$\mathrm{BR}=\frac{\max\;\left(|C\left(\hat{G}\right)|-|C\left(G\right)|,\;0\right)}{\left|C(G)\right|}.$$

Here, BR=0 indicates no excess fragmentation relative to the reference, whereas larger values indicate greater overfragmentation.

A complementary continuity ratio can be defined as the fraction of reference crack length recovered within tolerance:

(20) $${\mathrm{CR}}_{\tau }=\frac{L_{\tau }\left(G,\hat{G}\right)}{L(G)}.$$

Higher values of ${\mathrm{CR}}_{\tau }$ indicate that a larger fraction of the reference crack network is recovered as a spatially continuous structure. A ground-truth centerline element is considered matched when at least one predicted skeleton element lies within Euclidean distance τ after the declared skeletonization and registration procedure.

Skeleton-level precision and recall can then be written as

(21) $$P_{\tau }=\frac{L_{\tau }\left(\hat{G},G\right)}{L(\hat{G})},\;R_{\tau }=\frac{L_{\tau }\left(G,\hat{G}\right)}{L(G)},$$

with the corresponding skeleton-level F1 score

(22) $$F1_{\tau }=\frac{2P_{\tau }R_{\tau }}{P_{\tau }+R_{\tau }}.$$

As reported in prior studies, the interpretation of these measures depends on the skeletonization procedure, the matching tolerance τ, and whether evaluation is performed before or after corridor stitching; continuity results are, therefore, most comparable when those implementation details are declared explicitly.

### 7.5. Mapping Geometric Outputs to PCI/DIM

Studies that connect LiDAR measurements to agency practice typically do so through threshold-based severity categories defined on geometric quantities such as crack width, rut depth, or pothole depth [30,31]. Let *d* denote a measured geometric quantity and $T_{k}$ the boundary between severity classes *k* and k+1. Severity assignment can be written as

(23) $$S\left(d\right)=\left\{\begin{array}{cc} k & \mathrm{if}\;T_{k-1}\leq d<T_{k},\\ k+1 & \mathrm{if}\;T_{k}\leq d<T_{k+1}. \end{array}\right.$$

Within PCI-style workflows, severity and quantity are then translated into deduct values through non-linear lookup procedures [31]. As a result, small measurement deviations near a threshold may change the assigned severity class and, in turn, the final PCI outcome. In this sense, PCI-style interpretation differs from segmentation-style evaluation because the mapping from geometry to decision category is discontinuous.

### 7.6. Measurement Uncertainty and Threshold Sensitivity

The severity literature also shows that geometric measurement error must be interpreted in light of sensor noise, acquisition conditions, and reference-surface estimation. In LiDAR-based pavement analysis, uncertainty may arise from ranging noise, incidence-angle effects, surface reflectance variation, and trajectory error in mobile laser scanning systems [41,54]. After detrending and local reference-surface fitting, the estimated depth can be expressed as

(24) $$\hat{d}=d+\epsilon_{\mathrm{range}}+\epsilon_{\mathrm{plane}},$$

where $\epsilon_{\mathrm{range}}$ denotes sensor range noise and $\epsilon_{\mathrm{plane}}$ denotes residual error associated with local reference-surface estimation after detrending.

If measurement uncertainty is characterized by variance $\sigma_{d}^{2}$, then the risk of severity misclassification increases when the estimated depth lies near a threshold:

(25) $$|\hat{d}-T_{k}|\leq z\sigma_{d},$$

where *z* corresponds to the chosen confidence level.

Because PCI deduct functions are piecewise and non-linear [31], the corresponding PCI sensitivity can be written as

(26) ΔPCI≈f(S(d+δ))−f(S(d)),

where δ denotes a measurement perturbation and f(·) denotes the PCI deduct mapping. When *d* lies close to $T_{k}$, even a small perturbation may produce a non-zero change in PCI.

Related roughness standards also emphasize repeatability and tolerance-based reporting for stable interpretation [57]. In the same spirit, LiDAR-based distress studies are more informative when they report depth error statistics, tolerance-band compliance, and the sensitivity of severity assignments to threshold proximity. Taken together, the literature suggests that evaluation for corridor-scale LiDAR pavement assessment is most informative when overlap-based results are interpreted alongside geometric uncertainty and threshold sensitivity within PCI/DIM-style decision frameworks.

## 8. Challenges and Future Directions

Corridor-scale LiDAR surveys produce large data volumes, yet the corresponding labels are often sparse, uneven, and expensive to obtain, especially for thin structures such as cracks and narrow surface discontinuities [8,10,32,34,35]. Point-wise annotation, therefore, remains a major bottleneck for comparative evaluation and for the development of native 3D methods [8,10,11,32]. Label-efficient pipelines, geometry-driven grouping, reconstruction-based anomaly mapping, and semi-/weakly supervised approaches provide practical alternatives, but their evaluation is often constrained by narrow benchmark coverage and inconsistent reference data [10,18,27,28,29,80,81,82,83,84]. Public resources such as RSRD-Seg and recent handheld-LiDAR crack datasets represent useful steps forward, but the literature still points to a need for broader multiregion datasets with clearer severity definitions and stronger alignment with PCI/DIM-oriented reporting [30,31,34,35]. Future progress would benefit from annotation tools that operate natively on point clouds, protocols for confidence-rated pseudo-labels, and active-learning strategies tailored to thin, topological targets such as crack skeletons.

Generalization remains a persistent challenge in LiDAR-based pavement assessment. Studies conducted on one road type, climate, or acquisition regime do not necessarily transfer cleanly to another, and factors such as intensity normalization, detrending, and overlap-aware tiling are often introduced to improve stability across runs [2,9,13,21,22,26,40,41]. At the same time, cross-season, cross-region, and cross-platform evaluations remain relatively limited in the current literature [3,18,34,35,50,51]. Future work would benefit from geographically disjoint and seasonal splits, more explicit reporting of transfer performance, and domain-generalization or self-training strategies designed for sparse, elongated defects. Benchmark coverage would also be strengthened by greater attention to unsealed and gravel roads, where corrugation and settlement do not fit neatly into standard crack/pothole taxonomies.

The literature on RGB–LiDAR fusion suggests that realized gains depend strongly on calibration quality, synchronization, and common field of view rather than on sensor combination alone [2,9,13,21,22,26,91]. Small spatial misalignments can weaken correspondence between texture and geometry, and temporal skew can disrupt thin crack topology in fused outputs [2,9,13,21,22,26,91]. A further practical limitation is partial field-of-view overlap: camera imagery may cover only part of the pavement area sampled by LiDAR, so fusion is only meaningful within the common support of the two sensors. Accordingly, future fusion studies would be more informative if they reported alignment error, sensitivity to synthetic perturbations, per-stream versus fused performance, and fallback behavior when calibration quality degrades.

For infrastructure management, geometric measurements are often more actionable than masks alone because agency workflows ultimately depend on severity-linked quantities rather than only detection maps [30,31,38,57]. Reported outputs, therefore, need to preserve crack width and continuity, rut depth and profile spacing, and pothole depth or volume from locally detrended surfaces, together with clear unit conventions and uncertainty information [6,7,13,30,31,36,38,41,57]. When results are mapped to DIM/PCI-style categories, measurement error near severity thresholds can affect both class assignment and downstream deduct values [30,31]. Future work would be more directly useful for pavement management if segmentation scores were reported alongside metrology error, tolerance-band compliance, and confidence intervals.

The available literature and benchmark ecosystem still emphasize asphalt cracks and discrete potholes more than several other operationally important distress types [3,14,15,34,35,38,43]. Distresses such as edge failure, bleeding or flushing, raveling, gravel-road corrugation, and localized settlement receive less systematic treatment despite their practical relevance in field inspection and maintenance planning [30,31,38,43]. Additional datasets and task formulations targeted to these cases, such as periodicity-aware analysis for corrugation or edge-aware measures for shoulder deterioration, would broaden applicability and reduce deployment surprises.

Prior studies also suggest that profiler-style or lower-density LiDAR configurations can still support selected pothole-sizing and roughness-related tasks when detrending and basin or profile modeling are handled carefully, although the limits imposed by sparse sampling must be made explicit [7,13,36,38,49,57]. This points to a broader need for work that characterizes the trade-off between accuracy and throughput across sensor classes and that explores adaptive pipelines capable of switching between 2.5D and native 3D processing according to observed density, defect scale, and compute budget.

Deployment-oriented studies increasingly highlight the importance of explicit performance budgeting at corridor scale [15,46,92]. Across the literature, recurring implementation patterns include overlap-aware tiling, lightweight candidate generation, staged refinement, and separation between latency-sensitive screening and more computationally intensive back-end analysis [14,15,38,92]. To improve comparability, studies should co-report quality metrics (e.g., IoU/F1 or mAP) together with efficiency measures such as tile rate, end-to-end latency, hardware specification, and memory use [15,46,92]. Future work could also examine queue-aware inference, on-vehicle health monitoring, and practical visual inspection workflows for identifying calibration or coverage failures before results are ingested into inventory systems.

The review also reinforces that conventional IoU and F1 do not fully characterize thin-crack performance because they can obscure fragmentation of elongated structures [8,10,11,20,32,64,85,96,97]. Continuity-aware measures, such as break rate, continuity ratio, and skeleton-level matching, therefore, deserve broader use in crack-centric evaluation, particularly for point- and graph-based methods that aim to preserve connectivity [8,10,11,20,32,85,96,97]. For fusion studies, this argues for reporting both single-stream and fused baselines under identical splits and stated alignment tolerances; for reconstruction-oriented studies, it argues for reporting geometry error and completeness alongside detection performance.

Finally, several sections of the literature point to the importance of uncertainty-aware reporting rather than accuracy summaries alone [30,31,38,41,57]. Confidence estimates at both mask and measurement level, such as intervals on crack width or pothole depth, can, in principle, be propagated into PCI-related confidence bounds and can improve the interpretability of automated assessments for review workflows [30,31,38,57]. Coupling such outputs with calibration and timing diagnostics, including reprojection residuals or skew estimates where relevant, would further strengthen traceability in asset-management contexts.

Taken together, these observations suggest a practical minimum reporting set for future LiDAR-based pavement studies: platform and sensor configuration, speed and point density, synchronization and calibration procedure, tiling strategy, preprocessing choices, detrending and reference-surface definitions, task-specific evaluation metrics, and runtime characteristics. Where possible, reproducibility would also be improved by releasing code for stitching and severity computation and by defining official dataset splits that are geographically and temporally disjoint, with optional stress splits for season, pavement type, or sensor transfer.

### 8.1. Research Roadmap

While some of the architectural trends discussed below originate from broader point-cloud segmentation research, they are included here only insofar as they inform LiDAR-based pavement-distress analysis.

The literature reviewed in this survey suggests a practical development path for LiDAR-based pavement distress segmentation and measurement. Progress tends to depend less on novel architectures in isolation and more on (i) robustness to field conditions and acquisition variability, (ii) preservation of thin-structure continuity and severity-relevant geometry, and (iii) deployment constraints such as throughput, calibration drift, and auditing. Table 16 summarizes a three-stage roadmap aligned with these priorities.

**Stage I: Robustness under field acquisition variability** 

Early point-cloud segmentation methods developed for general 3D scene analysis established effective point-wise feature learning and neighborhood aggregation (e.g., PointNet-style pipelines and later point/voxel convolutions) [64,94]. Although these methods were not originally designed for pavement analysis, their architectural principles have influenced later LiDAR-based road surface segmentation pipelines. Pavement point clouds, nevertheless, present additional stressors, including strong density variation under motion, occlusions from traffic, severe foreground–background imbalance, and seasonal effects. Recent work on imbalance-aware learning explicitly targets long-tail and rare-class failure modes [98], which is directly relevant when crack and spall points constitute a small fraction of a corridor tile. Near-term emphasis should be on repeatable splits and cross-platform evaluation (e.g., MLS vs. handheld depth vs. ALS/UAV-LS where applicable), with preprocessing and sampling choices reported in a way that supports reproduction across sensors.

**Stage II: Structure- and severity-aware modeling** 

For pavement distress, continuity and geometry matter as much as per-point accuracy. Thin cracks can fragment into disconnected components even when overlap metrics remain acceptable. Skeletonization and thinning methods provide standard tools for extracting and evaluating centerline structures [96]. Likewise, the broader trend toward richer scene parsing objectives motivates reporting that distinguishes point-wise labeling from structural integrity and boundary faithfulness [97]. Mid-term work should, therefore, prioritize (i) continuity-aware objectives and evaluation (e.g., break rate, continuity ratio, skeleton-level matching), (ii) representation choices that preserve millimetric depth residuals needed for severity (width/depth/volume), and (iii) explicit treatment of reference-surface fitting and detrending assumptions when reporting severity error.

**Stage III: Deployment-oriented reliability and scalability** 

Corridor-scale operation imposes hard limits on latency, memory, and quality control. Methods that perform well in controlled settings can degrade under calibration drift, clock skew, or density loss; these effects are amplified for thin defects. Feature aggregation strategies developed in large-scale LiDAR segmentation research illustrate how multiscale context can be integrated efficiently [99]; analogous ideas are useful for road corridors when compute must be bounded. Longer-term work should focus on (i) stable edge–cloud partitioning for screening versus severity refinement, (ii) calibration/timing diagnostics and fallbacks that keep outputs auditable, and (iii) reporting that pairs accuracy with throughput and failure modes under controlled perturbations.

### 8.2. Recent Advances and Emerging Trends (2023–2025)

Recent work has increasingly shifted from reporting aggregate segmentation accuracy alone toward addressing practical failure modes relevant to pavement assessment, especially long-tail class imbalance, density variation, thin-structure continuity, and severity-aware evaluation [98,99,100]. In the recent literature, this shift appears in stronger attention to rare-class learning, multiscale feature aggregation under non-uniform sampling, and evaluation practices that extend beyond point-wise IoU/F1 to include continuity-sensitive and geometry-linked measures [98,99,100]. For this survey, the importance of these developments is not only that they improve segmentation performance, but that they align more closely with the operational needs of LiDAR-based pavement analysis, including crack continuity, depth reliability, and reproducible reporting across datasets and acquisition regimes. This recent shift reinforces the central argument of the present survey: LiDAR-based pavement analysis is moving from detection-oriented benchmarking toward geometry-aware, severity-aware, and deployment-relevant evaluation.

## 9. Conclusions

LiDAR has become an important modality for pavement-distress analysis because it provides direct access to surface geometry and supports depth-aware characterization under conditions where image-only methods may be unreliable. This survey reviewed LiDAR-based pavement analysis across the full processing pipeline, from acquisition and calibration through preprocessing, representation, modeling, deployment, and evaluation, and organized the literature using an eight-dimension synthesis framework.

Across the reviewed studies, a consistent theme is that effective pavement analysis depends on more than detection accuracy alone. Acquisition quality, calibration stability, preprocessing choices, and representation design all influence the geometric reliability of downstream measurements. This is especially important for severity-oriented tasks, where crack width, rut depth, pothole depth, and related quantities must remain meaningful under agency frameworks such as DIM and PCI.

The literature also shows that the field is advancing from detection-focused studies toward more geometry-aware, severity-aware, and deployment-conscious pipelines. At the same time, progress remains constrained by fragmented datasets, limited public benchmarks, inconsistent reporting, and weak comparability across sensing platforms and evaluation protocols.

Overall, LiDAR-based pavement analysis appears most promising when geometric fidelity, learning efficiency, and operational scalability are treated jointly rather than in isolation. Continued progress will depend on stronger benchmark resources, clearer metrological reporting, better support for cross-domain robustness, and tighter integration between automated outputs and infrastructure-management practice.

## Figures and Tables

**Table 1:** Task–output–representation–evaluation crosswalk for LiDAR-based pavement distress analysis. This table binds the survey structure by linking each task to typical outputs, representations, and evaluation focus.

Task	Operational Question	Typical Outputs	Typical Representations/ Model Families	Primary Evaluation Focus
Detection	“Is there distress here?” (screening)	Tile-level flags; pixel/point masks for cracks, potholes, ruts	2.5D grids/range images + 2D CNNs; native point/graph models; sparse voxels	IoU/mIoU, F1/Dice, PR curves; continuity-aware reporting for cracks
Inventory	“What types, how many, and where?” (instances + mapping)	Instances (potholes, crack segments, patches); corridor inventory layers	Instance extraction via CC/clustering/skeleton segments; overlap stitching; geo-aggregation	AP/mAP, counting error, localization/extent error; duplicate suppression under overlap tiling
Severity	“How bad is it?” (measurement + standards)	Width/depth/volume; rut profiles; ordinal bins; DIM/PCI-linked quantities	Detrending + reference surface; 3D measurement; regression/ordinal models	MAE/RMSE/bias and tolerance bands; volume error; DIM/PCI threshold sensitivity; IRI error (if reported)

**Table 3:** Comparative clarity of prior surveys under the task–output–representation–evaluation (TORE) crosswalk for LiDAR-based pavement-distress analysis. Legend: ● explicit and systematic coverage, ◗ partial or implicit coverage, ❍ minimal/absent.

Survey	Task	Output	Representation	Evaluation
[44]	◗	◗	◗	●
[3]	◗	◗	●	●
[14]	◗	◗	●	●
[15]	◗	◗	●	●
[16]	◗	◗	◗	●
[17]	◗	◗	◗	◗
[18]	◗	◗	◗	●
[19]	◗	◗	◗	●
[20]	◗	◗	◗	●
[23]	◗	◗	●	●
[24]	◗	◗	●	●
[42]	◗	◗	◗	●
[40]	◗	◗	●	◗
[41]	◗	◗	●	◗
[38]	❍	◗	◗	●
[43]	❍	◗	◗	●
[46]	◗	◗	◗	●
[45]	◗	◗	●	●
[47]	◗	◗	◗	◗
[39]	◗	◗	●	●
**This Survey**	●	●	●	●

**Table 4:** Search protocol used for literature retrieval.

Database	Representative Query	Year Filter	Result Count	Document Types
IEEE Xplore	LiDAR AND (pavement OR “road surface”) AND (crack OR rut OR pothole OR distress) AND detection	2015–2025	49	Journal articles/conference papers
ScienceDirect	LiDAR AND (pavement OR “road surface”) AND (crack OR rut OR pothole OR distress) AND detection	2015–2025	512	Research articles/review articles
SpringerLink	LiDAR AND (pavement OR “road surface”) AND (crack OR rut OR pothole OR distress) AND detection	2015–2025	142	Research articles/review articles
MDPI	LiDAR AND (pavement OR “road surface”) AND (crack OR rut OR pothole OR distress) AND detection	2015–2025	16	Articles/review articles

**Table 5:** Benchmark audit of dataset categories relevant to LiDAR-based pavement-distress analysis. The table emphasizes benchmark availability, sensing origin, task support, and reproducibility rather than only listing dataset names.

Category	Representative Resources	Sensing Origin	Tasks	Labels	Severity	Access	Reproducibility Implication
Public LiDAR-native pavement datasets	RSRD-Seg [34]	Mobile LiDAR + stereo	D; I (lim.); S (part.)	Dense/sparse surface labels	Part.	Y	One of the stronger currently available options, although still limited in geographic coverage and task diversity.
Limited-release LiDAR datasets	Apple LiDAR Pavement Cracks [35]	Handheld LiDAR	D; crack geom. (lim.)	Crack-level annotations	Lim.	L	Useful for exploratory comparison, but limited release and incomplete documentation restrict benchmark standardization.
Reconstructed-geometry datasets (non-LiDAR-native)	Road Surface 3D Reconstruction [48]	Stereo/RGB-D geometry	S; reconstruction	Geometry ground truth	Y	Y	Useful for geometric validation, but not representative of LiDAR-native acquisition conditions.
Private or study-specific LiDAR datasets	Multiple primary studies [7,13,36,37,41,49]	MLS, TLS, handheld, profiler-style, or custom setups	D; I; S	Study-dependent	Study-dependent	N	Support method development, but weak reproducibility, limited cross-paper comparability, and no stable public benchmark.
Auxiliary RGB/ multimodal transfer datasets	RDD variants [50,51], KITTI Road [52], Cityscapes [53]	RGB or generic multimodal	D; transfer; pretraining	Image-level or segmentation labels	N for LiDAR S	Y	Useful for transfer and baseline comparison, but not valid as LiDAR severity benchmarks.

*Abbreviations:* D = detection; I = inventory; S = severity; lim. = limited; part. = partial; Y = yes/public; L = limited or partial access; N = no/non-public.

**Table 6:** Core LiDAR-based pavement distress datasets used for geometry-aware detection and severity estimation.

Dataset	Region	Platform	Surface Type	Labels	Severity Validity	Seasonality	Public Access
RSRD-Seg [34]	China	Mobile LiDAR + stereo	Urban asphalt	Dense + sparse masks	Partial (reconstruction tasks)	Single-region acquisition	Yes
Apple LiDAR Pavement Cracks [35]	Unknown/ multiuser capture	Handheld LiDAR	Asphalt cracks	Crack-level annotations	Limited (geometry only)	Not reported	Partial
Road Surface 3D Reconstruction [48]	Unknown	Stereo/RGB-D geometry	Pothole surfaces	Geometry ground truth	Yes (depth/ volume)	Not reported	Yes

**Table 7:** Primary strengths and limitations of the representative LiDAR pavement datasets summarized in Table 6.

Dataset	Primary Strengths	Primary Limitations
RSRD-Seg [34] (2024)	LiDAR + stereo with calibrated motion (IMU/RTK); designed for fine-grained road surface geometry and reconstruction; supports both dense and sparse supervision.	Corridor-level coverage limited to the collection region and driving conditions; density depends on vehicle speed and fusion window; labels focus on road surface rather than diverse roadside context.
Apple LiDAR Pavement Cracks [35] (2025)	Enables low-cost close-range 3D crack capture; includes crack-type annotations; convenient for controlled comparisons across capture setups (handheld/gimbal/TLS).	Small spatial extent per sample; density/scale vary with capture distance and device settings; limited generalization to vehicle-mounted MLS/ALS scenarios.
Road Surface 3-D Reconstruction & Pothole Dataset [48] (2022)	Geometry-focused ground truth for 3D reconstruction and pothole analysis; useful for evaluating metric reconstruction accuracy.	Not LiDAR-native; limited scene variety and spatial extent vs. corridor-scale mobile LiDAR; performance depends on camera calibration and lighting.

**Table 8:** Auxiliary datasets frequently used for transfer learning, benchmarking, or multimodal comparison in pavement-damage research. These datasets provide useful baselines but do not contain LiDAR-based geometric measurements.

Dataset	Modality	Primary Use	Limitation for LiDAR Studies
RDD2020 [50]	RGB	Damage detection benchmarking	No geometric depth or severity measurement
RDD2022 [50]	RGB	Large-scale classification benchmark	Image-only distress representation
N-RDD 2024 [51]	RGB	Expanded damage taxonomy	No geometric surface information
KITTI Road [52]	LiDAR + stereo	Road segmentation baseline	Not annotated for pavement distress
Cityscapes [53]	RGB	Urban scene pretraining	No pavement damage annotations

**Table 9:** Coverage of recommended minimum metadata categories for representative datasets. Legend: ● documented; ◗ partially documented or dataset-dependent; ❍ not documented or not applicable.

Dataset	Sensor Model & Scan Pattern	Nominal Ranging Accuracy	Ground Point Spacing	Intensity Calibration Status	Georeferencing Method	Ground-Truth Generation
RSRD-Seg (2024) [34]	◗	◗	◗	◗	●	●
Apple LiDAR Pavement Cracks (2025) [35]	◗	❍	◗	◗	❍	●
Road Surface 3D Reconstruction (2022) [48]	◗	❍	◗	❍	◗	●

**Table 10:** Task support level of representative datasets reviewed in this survey. “Detection” refers to mask-level evaluation; “Inventory” to instance-level evaluation; “Severity (Regression)” to geometric measurement validation; “Severity (Ordinal)” to bin-based or DIM/PCI-aligned labels.

Dataset	Detection	Inventory	Severity (Regression)	Severity (Ordinal)
RSRD-Seg (2024) [34]	**✓**	Limited	Partial	**✗**
Apple LiDAR Pavement Cracks (2025) [35]	**✓**	**✗**	Limited	**✗**
Road Surface 3D Reconstruction (2022) [48]	Limited	**✗**	**✓**	**✗**
RDD variants [50,51]	**✓**	**✓**	**✗**	Limited
Profiler/IRI-based datasets [38,57]	**✗**	Limited	**✓**	**✗**

*Legend: ***✓** = direct task support; Limited = indirect or incomplete support; Partial = support for some but not all required outputs or labels; **✗** = no explicit support.

**Table 11:** LiDAR data representations.

Representation	Typical Channels/Features	Best-Suited Tasks	Common Model Families	Notes
2.5D rasters	height, slope, roughness, intensity, normal	Screening; fast inventory	U-Net/FPN, ViT-seg	Simple training; loses fine vertical relief
Range image	range, remission, return count, curvature	Screening; scan-aligned inventory	2D CNN/U-Net (circular pad)	Efficient; requires reprojection for 3D metrics
Native 3D	xyz, intensity, normal; kNN graph or sparse voxels	Geometry & severity (width/depth/volume)	PointNet++/KPConv/ RandLA; Minkowski 3D U-Net; GNNs	Highest geometric fidelity; heavier compute

**Table 12:** Architectural comparison with task-dependent limitations for pavement analysis.

Architecture	Strengths	Limitations (Task-Dependent)	Most Appropriate Tasks
2.5D CNN/U-Net [60]	Efficient; mature 2D segmentation frameworks; compatible with raster workflows.	Grid resolution limits crack detectability if cell size exceeds defect width; projection smoothing may bias depth residuals; limited suitability for volumetric severity without auxiliary geometric modeling.	Detection; coarse inventory mapping
Range Image CNN [62]	Computationally efficient; leverages spherical structure of rotating LiDAR.	Sensitive to sensor-specific angular resolution; domain shift across LiDAR models; limited geometric fidelity for precise depth estimation.	Detection; real-time applications
PointNet/PointNet++ [61,64]	Preserves raw geometry; multiscale neighborhood abstraction.	Neighborhood radius selection may oversmooth thin cracks; computational cost increases with point density; sensitive to extreme density variation.	Severity estimation; fine-grained segmentation
Graph Convolutional Networks [67,68]	Explicitly models local topology; improves crack continuity.	Graph construction cost scales with point count; memory overhead; performance sensitive to noisy edges in sparse regions.	Detection with continuity preservation; inventory mapping
Sparse 3D Convolutions [65,66]	Scalable to large scenes; efficient sparse computation.	Voxel quantization suppresses thin defects if resolution is coarse; memory increases with finer voxels; depth precision tied to voxel size.	Large-scale detection; corridor-level screening
Hybrid Point–Voxel	Balances efficiency and geometric precision; staged refinement.	Pipeline complexity; early detection errors may propagate to severity stage; tuning required for stable refinement.	Detection followed by volumetric severity estimation

**Table 13:** Common imbalance-aware objectives used for thin-structure detection and their typical effects.

Objective	Primary Benefit (Detection)	Typical Failure Mode/Caution
Focal [71]	Down-weights easy negatives; focuses learning on hard pixels/points	Sensitive to label noise; requires tuning (α,γ)
Dice [72]	Overlap-sensitive; less sensitive to class frequency	Unstable gradients for extremely small targets without smoothing ϵ
Generalized Dice [73]	Explicit rare-class reweighting; improves recall under severe imbalance	Can increase false positives if labels are noisy or boundaries are ambiguous
Tversky/Focal-Tversky [74]	Controls FP/FN tradeoff; supports recall-focused crack recovery	Poorly chosen (α,β) can bias toward oversegmentation
Lovász-Softmax [75]	Direct surrogate for IoU; improves rare-class IoU	Benefits depend on batching and label quality
Boundary/topology terms [76,77]	Improves edge fidelity and connectivity of thin structures	Often needs combination with region loss; sensitive to boundary noise
Class-balanced weights [78]	Principled weighting using effective sample counts	Choice of β controls aggressiveness; may still need patch sampling

**Table 14:** Survey-level qualitative synthesis of RGB–LiDAR fusion design trade-offs in pavement detection. The table summarizes commonly reported tendencies in the literature rather than a controlled cross-study benchmark, because published results differ in datasets, calibration quality, and evaluation protocols.

Fusion Level	Typical Benefit	Common Limitation	When Most Effective
Early	Strong joint feature interaction; potential highest peak IoU	Highly sensitive to spatial misalignment	Stable calibration; controlled acquisition
Mid-level	Improved robustness to minor misalignment	Increased memory/latency cost	Moderate calibration uncertainty
Late	Reduced false positives via agreement rules	Limited cross-modal interaction	Heterogeneous sensing or drift-prone systems

**Table 15:** Compact glossary of evaluation metrics discussed in this survey.

Metric Family	Typical Use	Primary Limitation in Pavement LiDAR
IoU/mIoU/Dice/F1	Detection and semantic segmentation	Can obscure crack fragmentation and structural discontinuity
AP/mAP	Inventory and instance detection	Sensitive to instance definition, stitching, and duplicate suppression rules
Counting/localization error	Corridor inventory	Depends strongly on aggregation unit and overlap reconciliation policy
MAE/RMSE/bias	Severity regression	Not comparable across datasets without aligned reference-surface and calibration assumptions
Tolerance-band compliance	Severity metrology	Requires declared unit calibration and independent reference measurements
Ordinal agreement/class accuracy	DIM/PCI-style severity bins	Sensitive to threshold proximity; can hide measurement instability near boundaries
IRI error	Roughness/profile validation	Depends on profile extraction, filtering, and profiler-standard assumptions
Continuity metrics	Crack-network structure	Require explicit centerline/skeleton extraction and matching tolerance

**Table 16:** Proposed research roadmap for LiDAR-based pavement distress segmentation and measurement.

Stage	Research Focus
I	Robustness to acquisition variability; imbalance-aware training and sampling; cross-sensor evaluation and reporting consistency [64,94,98].
II	Continuity- and severity-aware modeling; topology/centerline evaluation; explicit depth/geometry measurement assumptions and error reporting [96,97].
III	Scalable deployment: throughput-aware inference, edge–cloud partitioning, calibration/timing monitoring, and auditable reporting under drift and density loss [99].

## Data Availability

No new data were created or analyzed in this study. Data sharing is not applicable to this article.

## Abbreviations

The following abbreviations are used in this manuscript:

Acq.	Acquisition
AI	Artificial Intelligence
ALS	Airborne Laser Scanning
ASTM	American Society for Testing and Materials
Cal./Reg.	Calibration/Registration
CNN	Convolutional Neural Network
Distrib.	Distribution
DL	Deep Learning
FHWA	Federal Highway Administration
FPN	Feature Pyramid Network
GCN	Graph Convolutional Network
GNSS	Global Navigation Satellite System
IMU	Inertial Measurement Unit
IRI	International Roughness Index
KITTI	Karlsruhe Institute of Technology and Toyota Technological Institute Dataset
LTPP	Long-Term Pavement Performance
MAE	Mean Absolute Error
ML	Machine Learning
MLS	Mobile Laser Scanning
Optim.	Optimization
PCI	Pavement Condition Index
Post/Quant.	Post-processing/Quantification
Prep.	Preprocessing
RDD	Road Damage Detection
RGB	Red, Green, Blue
RMS	Root Mean Square
RMSE	Root Mean Square Error
ROI	Region of Interest
RSRD	Road Surface Roughness Detection
TLS	Terrestrial Laser Scanning
UAV	Unmanned Aerial Vehicle
